# Pregnancy complications and new-onset maternal autoimmune disease

**DOI:** 10.1093/ije/dyae115

**Published:** 2024-08-27

**Authors:** Natalie V Scime, Sonia M Grandi, Joel G Ray, Cindy-Lee Dennis, Mary A De Vera, Hailey R Banack, Simone N Vigod, Alexa Boblitz, Hilary K Brown

**Affiliations:** Department of Health and Society, University of Toronto Scarborough, Toronto, Ontario, Canada; ICES, Toronto, Ontario, Canada; Child Health Evaluative Sciences, The Hospital for Sick Children, Toronto, Ontario, Canada; Dalla Lana School of Public Health, University of Toronto, Toronto, Ontario, Canada; ICES, Toronto, Ontario, Canada; Dalla Lana School of Public Health, University of Toronto, Toronto, Ontario, Canada; Li Ka Shing Knowledge Institute, St Michael’s Hospital, Toronto, Ontario, Canada; Li Ka Shing Knowledge Institute, St Michael’s Hospital, Toronto, Ontario, Canada; Lawrence S. Bloomberg Faculty of Nursing, University of Toronto, Toronto, Ontario, Canada; Faculty of Pharmaceutical Sciences, University of British Columbia, Vancouver, British Columbia, Canada; Collaboration for Outcomes Research and Evaluation, University of British Columbia, Vancouver, British Columbia, Canada; Centre for Health Evaluation & Outcome Science, St. Paul’s Hospital, Vancouver, British Columbia, Canada; Dalla Lana School of Public Health, University of Toronto, Toronto, Ontario, Canada; ICES, Toronto, Ontario, Canada; Department of Psychiatry, University of Toronto, Toronto, Ontario, Canada; Women’s College Research Institute, Women’s College Hospital, Toronto, Ontario, Canada; ICES, Toronto, Ontario, Canada; Department of Health and Society, University of Toronto Scarborough, Toronto, Ontario, Canada; ICES, Toronto, Ontario, Canada; Dalla Lana School of Public Health, University of Toronto, Toronto, Ontario, Canada; Women’s College Research Institute, Women’s College Hospital, Toronto, Ontario, Canada

**Keywords:** Autoimmune diseases, pregnancy complications, cohort studies, mothers, female, Ontario

## Abstract

**Background:**

Autoimmune diseases disproportionately impact women and female-specific aspects of reproduction are thought to play a role. We investigated the time-varying association between pregnancy complications and new-onset autoimmune disease in females during the reproductive and midlife years.

**Methods:**

We conducted a population-based cohort study of 1 704 553 singleton births to 1 072 445 females in Ontario, Canada (2002–17) with no pre-existing autoimmune disease. Pregnancy complications were preeclampsia, stillbirth, spontaneous preterm birth and severe small for gestational age (SGA). Royston-Parmar models were used to estimate the time-varying association between pregnancy complications and a composite of 25 autoimmune diseases from date of delivery to date of autoimmune disease diagnosis or censoring at death, loss of health insurance, or 31 March 2021. Models were adjusted for baseline socio-demographics, parity and comorbidities.

**Results:**

At 19 years (median = 10.9 years of follow-up), cumulative incidence of autoimmune disease was 3.1% in those with a pregnancy complication and 2.6% in those without complications. Adjusted hazard ratio (AHR) curves as a function of time since birth were generally L-shaped. Universally, risks were most elevated within the first 3 years after birth [at 1 year: preeclampsia AHR 1.22, 95% confidence interval (CI) 1.09–1.36; stillbirth AHR 1.36, 95% CI 0.99–1.85; spontaneous preterm birth AHR 1.30, 95% CI 1.18–1.44; severe SGA AHR 1.14, 95% CI 0.99–1.31] and plateaued but remained elevated thereafter.

**Conclusions:**

Prior history of pregnancy complications may be an important female-specific risk factor to consider during clinical assessment of females for possible autoimmune disease to facilitate timely detection and treatment.

Key Messages

- This population-based study of over 1.7 million births found that pregnancy complications were associated with modestly elevated incidence of maternal autoimmune disease in an L-shaped manner for up to 19 years following delivery.
- The greatest elevation in risks was observed for preeclampsia, stillbirth and spontaneous preterm birth within the first 3 years following delivery.
- Findings signal the need for greater healthcare provider awareness of pregnancy complications as a female-specific risk factor for autoimmune disease to facilitate timely detection and treatment of these debilitating chronic conditions.

## Introduction

Autoimmune diseases affect 7–10% of the population and are a leading cause of morbidity and premature mortality in young and middle-aged women.^1^^,^^2^ These chronic conditions arise from a damaging immune response directed at the body’s own tissues, and typically occur in biologically susceptible individuals after an immune-activating environmental trigger.^3^ Autoimmune diseases are broadly classified as systemic (e.g. systemic lupus erythematosus) or organ-specific (e.g. Grave’s disease targeting the thyroid gland). Sex disparities are well-established; females represent up to 80% of autoimmune disease cases and the sex ratio reaches 9:1 for certain diseases during the reproductive years.^2^^,^^4^ Although the basis for this disparity is unresolved, female-specific aspects of reproductive biology including pregnancy-related events are thought to play an underlying role.^5^

Preeclampsia, stillbirth, spontaneous preterm birth and fetal growth restriction collectively affect 15% of women and are associated with increased long-term risk of chronic diseases as women age.^6^^,^^7^ These pregnancy complications are multi-factorial in origin but share abnormal immune and inflammatory features,^8^^,^^9^ that suggest a possible link with subsequent autoimmune disease. Two systematic reviews reported an association between pregnancy complications and subsequent diagnosis of autoimmune disease in women^10^^,^^11^; however, included studies were often limited by small or homogenous samples, possible self-report recall bias and suboptimal confounding control.^10^^,^^11^ Moreover, possible variations in this association over time have been suggested,^12^^,^^13^ but not thoroughly investigated. The incidence of autoimmune disease peaks postpartum and rises again near midlife,^14^^,^^15^ and perinatal events may exert different effects over time which could be strongest in the initial post-pregnancy years.

Evidence on pregnancy complications and risk of autoimmune disease in women can advance our knowledge of female-specific aetiology of autoimmune disease and help identify high-risk groups. Therefore, this study investigated the time-varying association between pregnancy complications and new-onset autoimmune disease in females during the reproductive and midlife years.

## Methods

### Study design

We conducted a retrospective population-based cohort study in Ontario, the largest province in Canada with ∼140 000 births annually. Under a public healthcare system, all medically necessary health services are delivered to residents at no direct cost. We accessed and analysed health administrative datasets at ICES (Toronto), an independent, non-profit research institute whose legal status under Ontario’s health information privacy law allows it to collect and analyse healthcare and demographic data, without consent, for health system evaluation and improvement. The study was approved by the University of Toronto Ethics Board (No. 43489).

Obstetrical delivery records were deterministically linked (i.e. exact linkage on a single unique encoded identifier)^16^ with demographic and health services databases (Supplementary Table S1, available as Supplementary data at *IJE* online). Deliveries after 1 April 2012 were additionally linked to the Better Outcomes Registry & Network (BORN) Ontario dataset for prenatal health data. ICES data are valid and complete; 85% of primary hospital discharges have exact agreement with re-abstraction,^17^ including high sensitivity and specificity (>85%) of obstetrical data,^18^ and most BORN variables have >90% agreement with patient charts.^19^^,^^20^

### Study population

We included females aged 15–54 years hospitalized for ≥1 births of a singleton liveborn or stillborn infant ≥20 weeks gestation between 1 April 2002 and 31 March 2017, and who were eligible for Ontario health insurance 2 years before conception and up to delivery. We excluded females with missing variables (1.4%) and with pre-existing autoimmune disease (6.1% based on Outcomes defined below; Supplementary Figure S1, available as Supplementary data at *IJE* online). Dates of conception were estimated by subtracting gestational age determined by first or second-trimester ultrasonography, the gold standard for pregnancy dating^21^ experienced by >95% of Ontario mothers,^22^^,^^23^ from delivery dates.^22^

### Exposures

Pregnancy complications were preeclampsia, stillbirth, spontaneous preterm birth and severe small for gestational age (SGA) and were not mutually exclusive (Supplementary Table S2, available as Supplementary data at *IJE* online). Preeclampsia and its progressive forms [eclampsia, HELLP (Hemolysis, Elevated Liver enzymes and Low Platelets) syndrome] were measured using healthcare visits between 20 weeks gestation and delivery. Stillbirth, spontaneous preterm birth (labour onset and delivery <37 weeks without induction or Caesarean),^24^ and severe SGA (<5th sex- and age-specific birthweight percentile) as a proxy of fetal growth restriction^25^ were measured from the delivery record. In sub-type analyses, preeclampsia timing was classified as early- (<34 weeks) or late-onset (≥34 weeks)^26^; stillbirth type was classified with or without fetal congenital anomalies^27^; spontaneous preterm birth timing was classified as extremely to very (<28 to <32 weeks) or moderate to late (32 to <37 weeks) and type was classified as with or without preterm premature rupture of the membranes (PPROM)^28^; and SGA severity was classified as <3rd percentile or 3rd to <5th percentile.

### Outcomes

The primary outcome was a composite of 25 autoimmune diseases (including 15 female-predominant diseases^2^^,^^5^^,^^12^) measured by diagnostic codes in ≥1 hospitalizations or emergency department visits (Supplementary Table S3, available as Supplementary data at *IJE* online). We considered autoimmune disease as a group given their frequent co-occurrence and shared etiopathologies.^2^^,^^29^ We used acute care encounters since physician visit codes contain 3 digits and are too broad to distinguish many autoimmune diseases.^30^ This approach prioritized specificity over sensitivity, as is preferred for aetiologic research, by capturing more severe disease presenting in a hospital setting across a comprehensive range of diseases.

The secondary outcomes were individual female-predominant autoimmune diseases measured with diagnostic code algorithms validated against medical records that span outpatient physician visits, hospitalizations and emergency department visits: coeliac disease (sensitivity 84%, specificity 97%),^31^ multiple sclerosis (sensitivity 84%, specificity 100%),^32^ rheumatoid arthritis (sensitivity 78%, specificity 100%),^33^ and systemic autoimmune rheumatic diseases (SARD, i.e. systemic lupus erythematosus, scleroderma, Sjögren’s syndrome, polymyositis/dermatomyositis; sensitivity >80%, specificity >70%).^34^^,^^35^ This approach improved the validity of outcome measurement by capturing disease presenting in all healthcare settings and enabled us to generate some condition-specific insights.

### Covariates

Covariates were predetermined and measured for each contributing birth. Maternal demographics were age, parity, neighbourhood income quintile (based on postal code-linked Census data) and rural residence (community population <10 000) at delivery. Comorbidities within 2 years before conception were unstable and stable chronic medical conditions as an aggregate marker of comorbidity burden measured using the Johns Hopkins Adjusted Clinical Groups^®^ system (version 10.0)^36^^,^^37^; and pre-existing mental illness.^38^^,^^39^ We also measured antepartum haemorrhage. We did not measure past pregnancy complications to avoid inducing model bias given the complex relationships with subsequent childbearing and complication recurrence.^40^

For a sub-cohort with BORN data, we also measured prenatal factors including mode of conception (unassisted conception with or without an outpatient visit for infertility within 2 years before conception, or assisted conception),^41^ maternal smoking and pre-pregnancy obesity (body mass index ≥30 kg/m^2^ or a healthcare visit containing an obesity code within 2 years before conception).

### Statistical analyses

We compared baseline characteristics of females with and without ≥1 pregnancy complications using standardized differences. We used Royston-Parmar models, a form of flexible parametric survival analysis, to estimate the time-varying association between each pregnancy complication (modelled with a binary indicator) and incidence of maternal autoimmune disease with a robust variance estimator to account for multiple births to the same woman. We used restricted cubic splines to model the baseline hazard and time-varying effect with 3 and 2 internal knots, respectively.^42^ We counted person-time at risk in days from the date of delivery to the date of autoimmune disease diagnosis or censoring at death, loss of Ontario health insurance, or 31 March 2021. The at-risk population was all births for preeclampsia, stillbirth and spontaneous preterm birth, and only live births for severe SGA. We first estimated the cumulative incidence of autoimmune disease with 95% CIs by exposure group, followed by crude and adjusted hazard ratios (aHR) and 95% CIs controlling for year of cohort entry, maternal age, parity, income quintile, rurality and comorbidities. We also estimated adjusted incidence rate differences (IRD) and 95% CIs to contextualize public health relevance.

Several additional analyses were performed for our primary outcome. We explored whether results differed by exposure sub-type or when limiting the outcome to female-predominant autoimmune diseases. We also conducted four analyses to address potential outcome misclassification, effect modification and residual confounding. First, we excluded autoimmune thyroid diseases since they may shortly resolve following perinatal onset.^43^ Second, we implemented a washout period by excluding autoimmune diseases diagnosed within 365-day postpartum to reduce any delayed or transient diagnoses of autoimmune symptoms arising during pregnancy. Third, we stratified the results by antepartum haemorrhage to explore for effect modification from clinical events that increase the potential entry of fetal blood into the maternal circulation^44^; and by parity to explore effect modification by childbearing history. Fourth, we repeated our main models in the BORN sub-cohort with additional adjustment for mode of conception, maternal smoking and pre-pregnancy obesity. Owing to low cell counts, we did not analyse stillbirth sub-type or effect modification. Statistical analyses were performed in Stata MP version 16 and figures were generated in R version 3.6.1.

## Results

### Baseline characteristics

The cohort included 1 704 553 births to 1 072 445 females and a sub-cohort of 550 722 births to 285 836 females in BORN (Supplementary Figure S1, available as Supplementary data at *IJE* online). Overall, 12.5% of births were affected by ≥1 of the pregnancy complications of interest; the prevalence of preeclampsia, stillbirth, spontaneous preterm birth and severe SGA were 4.2%, 0.3%, 4.9% and 4.0%, respectively. Compared with births without pregnancy complications, affected births were more often to mothers who were primiparous, experienced antepartum haemorrhage and had stable chronic medical conditions (Table 1).

**Table 1.** Baseline characteristics of women delivering a singleton live birth with or without one or more pregnancy complications

Characteristic	No pregnancy complications		≥1 pregnancy complication		**Standardized difference** ^a^
	*n*	%	*n*	%	
**Full cohort**	**1 490 829**		**213 724**		
Maternal age					
15–24 years	235 379	15.8	39 042	18.3	0.07
25–34 years	943 089	63.3	128 424	60.1	0.07
35–44 years	310 021	20.8	45 656	21.4	0.01
45–54 years	2 340	0.2	602	0.3	0.03
Neighbourhood income quintile					
1 (low)	314 994	21.1	51 084	23.9	0.07
2	294 182	19.7	44 383	20.8	0.03
3	307 125	20.6	43 862	20.5	0.00
4	318 204	21.3	42 513	19.9	0.04
5 (high)	256 324	17.2	31 882	14.9	0.06
Rural place of residence	155 901	10.5	21 336	10.0	0.02
Parity					
Primiparous	624 688	41.9	117 024	54.8	0.26
Multiparous	866 141	58.1	96 700	45.2	0.26
Comorbidities					
Chronic unstable medical condition	156 813	10.5	25 135	11.8	0.04
Chronic stable medical condition	342 817	23.0	57 661	27.0	0.09
Mood, anxiety or psychotic disorder	201 395	13.5	33 657	15.7	0.06
Substance-related disorder	14 136	0.9	3 573	1.7	0.06
Antepartum haemorrhage	16 382	1.1	5 485	2.6	0.11
**BORN sub-cohort**	**481 431**		**69 291**		
Mode of conception					
Unassisted	423 140	87.9	59 109	85.3	0.08
Unassisted, prior infertility visit	46 376	9.6	7 787	11.2	0.05
Assisted	11 915	2.5	2 395	3.5	0.06
Maternal smoking	49 610	10.3	10 532	15.2	0.15
Pre-pregnancy obesity	77 650	16.1	14 424	20.8	0.12

BORN, Better Outcomes Registry & Network.

a Standardized differences >0.10 were considered clinically meaningful.

### Main analyses

**Figure 1.**
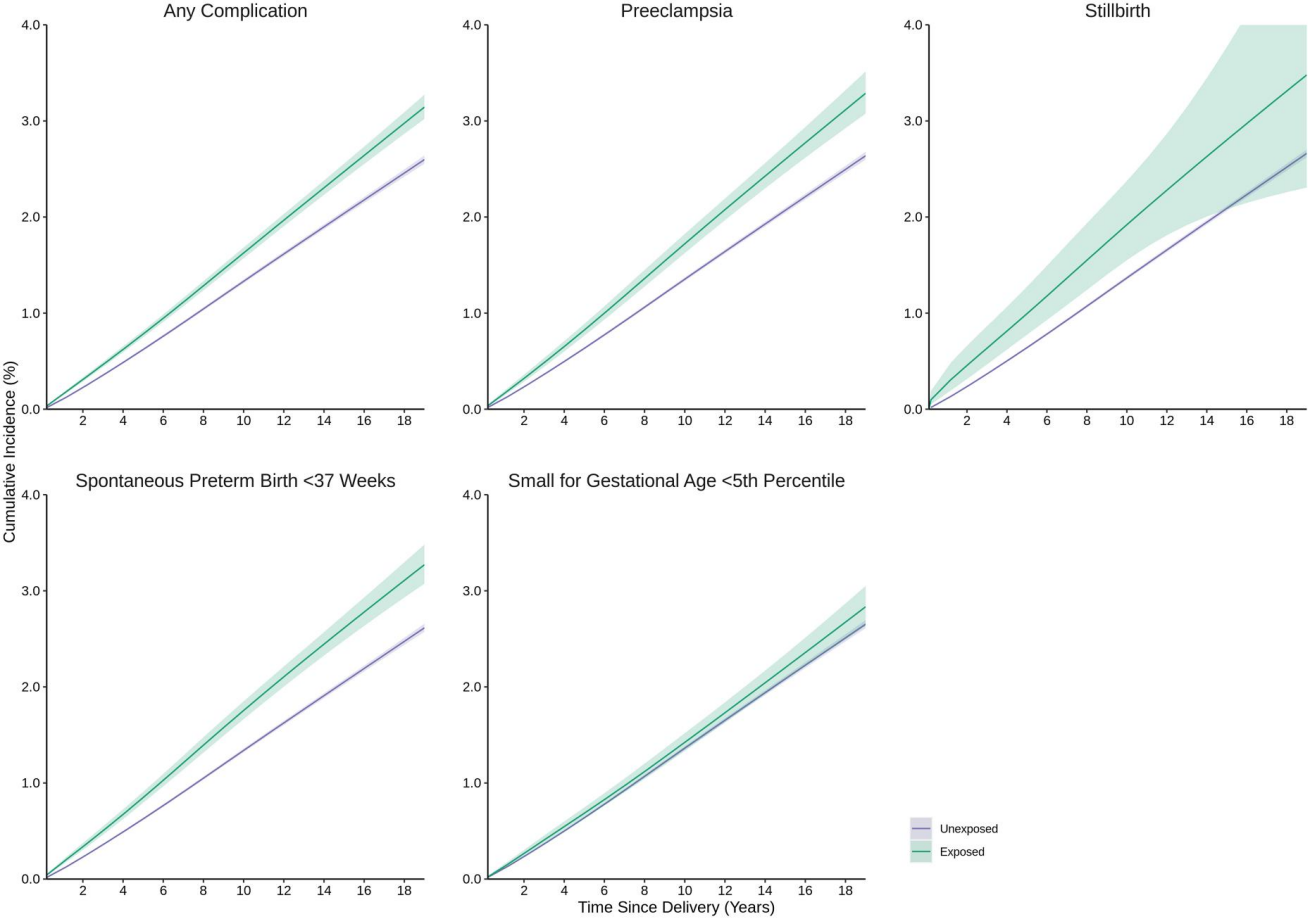
Cumulative incidence of autoimmune disease according to pregnancy complications. Autoimmune diseases for the composite primary outcome were measured using acute care (i.e. hospital-based) encounters only

**Figure 2.**
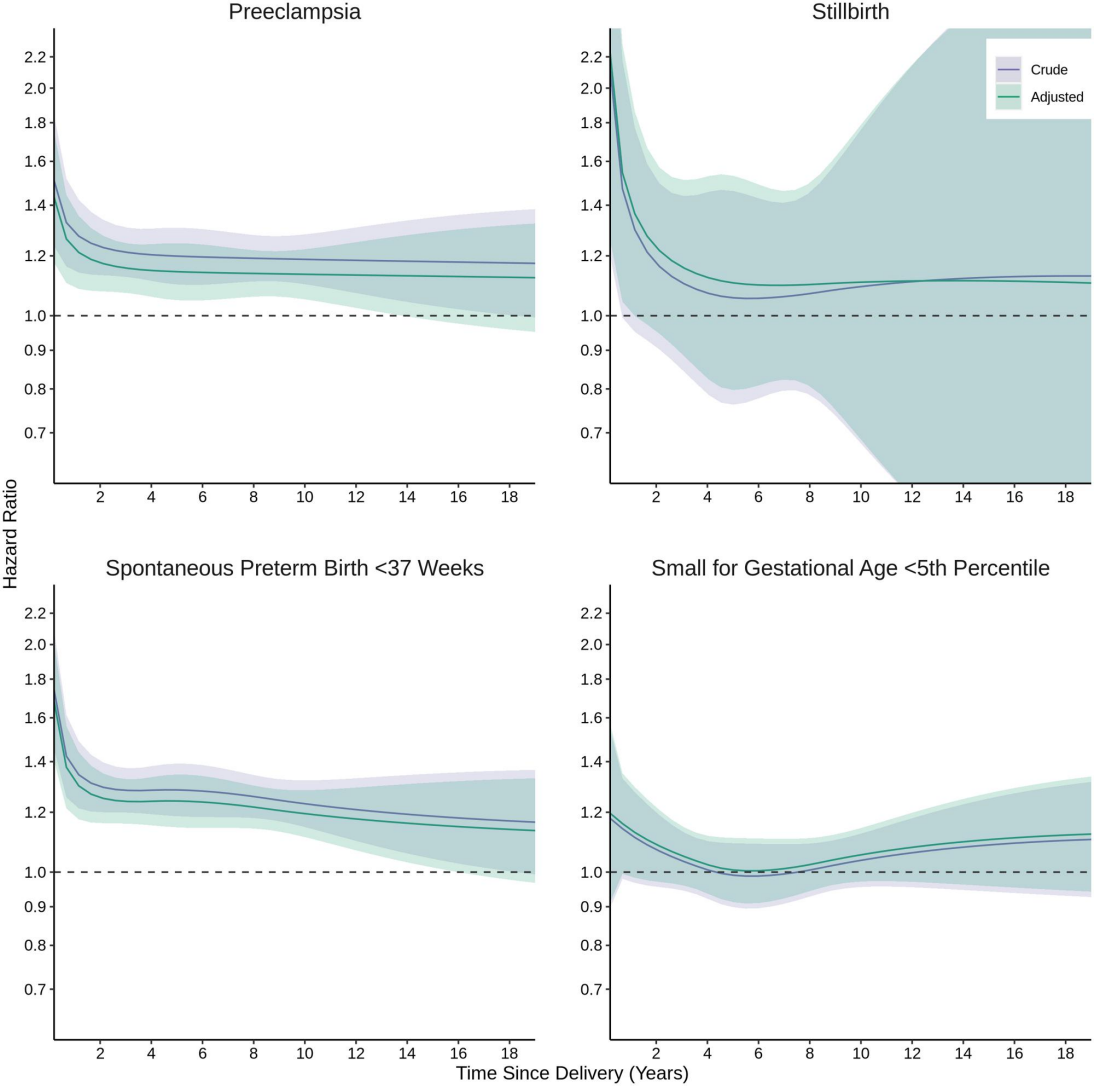
Time-dependent association of pregnancy complications and incident autoimmune disease. Adjusted models controlled for calendar year, maternal age at delivery, parity, neighbourhood income quintile, rural residence and medical and psychiatric comorbidities

At 19 years following birth [median = 10.9 years of follow-up per birth (interquartile range = 7.2–14.8 years)], the cumulative incidence of autoimmune disease was 3.1% (95% CI 3.0–3.3%) in those with ≥1 pregnancy complications and 2.6% (95% CI 2.5–2.6%) in those without (Figure 1). Certain pregnancy complications were associated with autoimmune disease in a time-dependent manner with generally L-shaped aHR curves as a function of time since birth; covariate adjustment slightly attenuated these associations (Figure 2, Supplementary Table S4, available as Supplementary data at *IJE* online). For preeclampsia, aHRs were largest between birth and 3 years (aHR, 95% CI at 1 year: 1.22, 1.09–1.36) and plateaued but remained elevated thereafter (5 years: 1.14, 1.05–1.25; 10 years: 1.13, 1.05–1.22). For stillbirth, aHRs were largest between birth and 1 year (1 year: 1.36, 0.99–1.85) and plateaued thereafter with increasingly degrading precision (5 years: 1.10, 0.80–1.53). For spontaneous preterm birth, aHRs were largest between birth and 2 years (1 year: 1.30, 1.18–1.44), and plateaued but remained elevated thereafter (5 years: 1.24, 1.15–1.35; 10 years: 1.20, 1.12–1.28). Severe SGA was not associated with risk of autoimmune disease; aHRs between birth and 2 years (1 year: 1.14, 0.99–1.31) as well as 14 and 19 years (15 years: 1.11, 0.96–1.28) were slightly elevated with a U-shaped parabola, but 95% CIs included the null. These results translated to small incidence rates and adjusted IRDs given the rarity of autoimmune disease (Supplementary Table S5, available as Supplementary data at *IJE* online).

**Figure 3.**
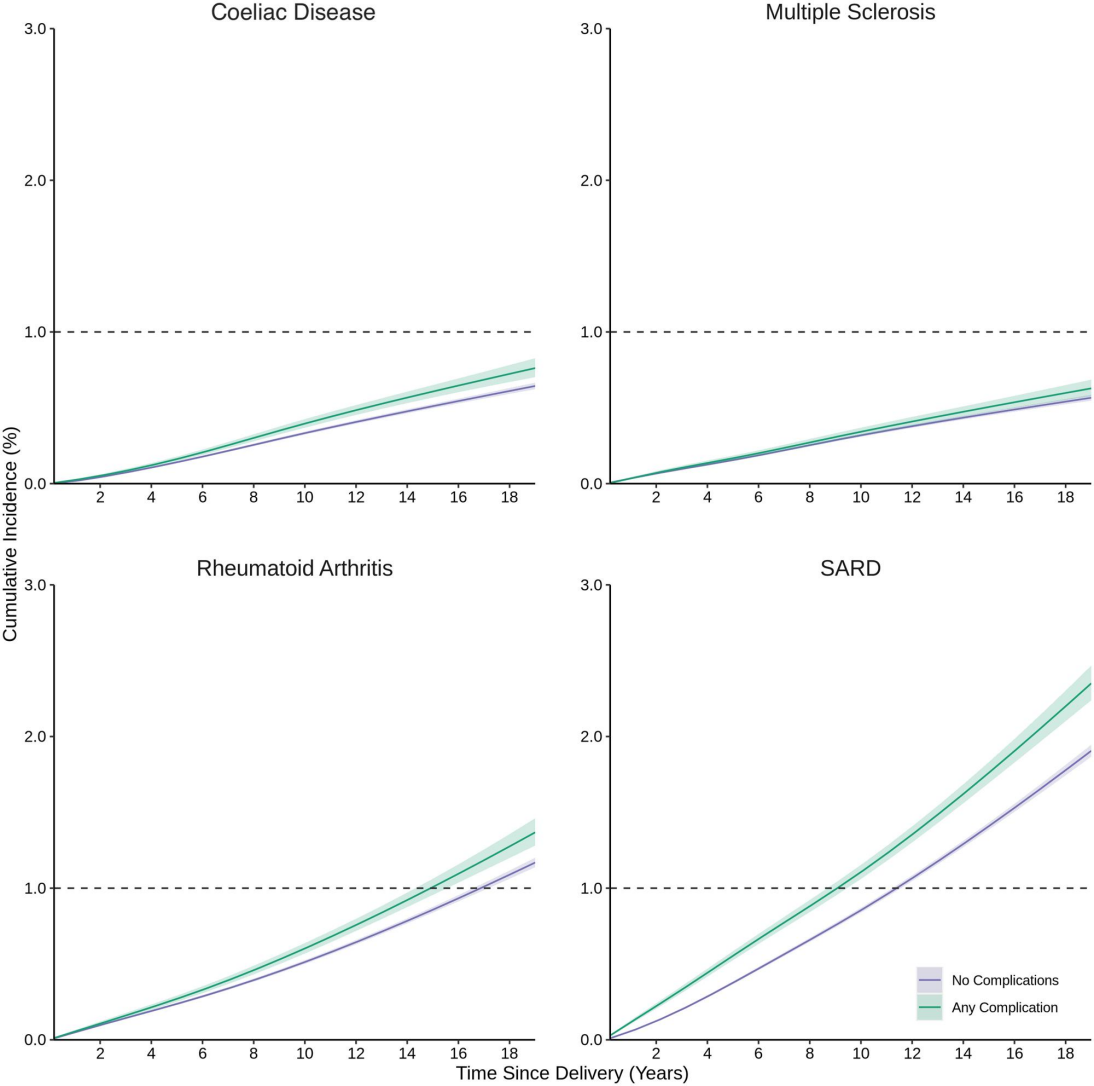
Cumulative incidence of individual female-predominant autoimmune diseases according to pregnancy complications. SARD: systemic autoimmune rheumatic disease. Any complication was defined as ≥1 of preeclampsia, stillbirth, spontaneous preterm birth <37 weeks or severe small for gestational age <5th percentile. Individual autoimmune diseases were measured using validated algorithms for acute care (i.e. hospital-based) and outpatient physician encounters

**Figure 4.**
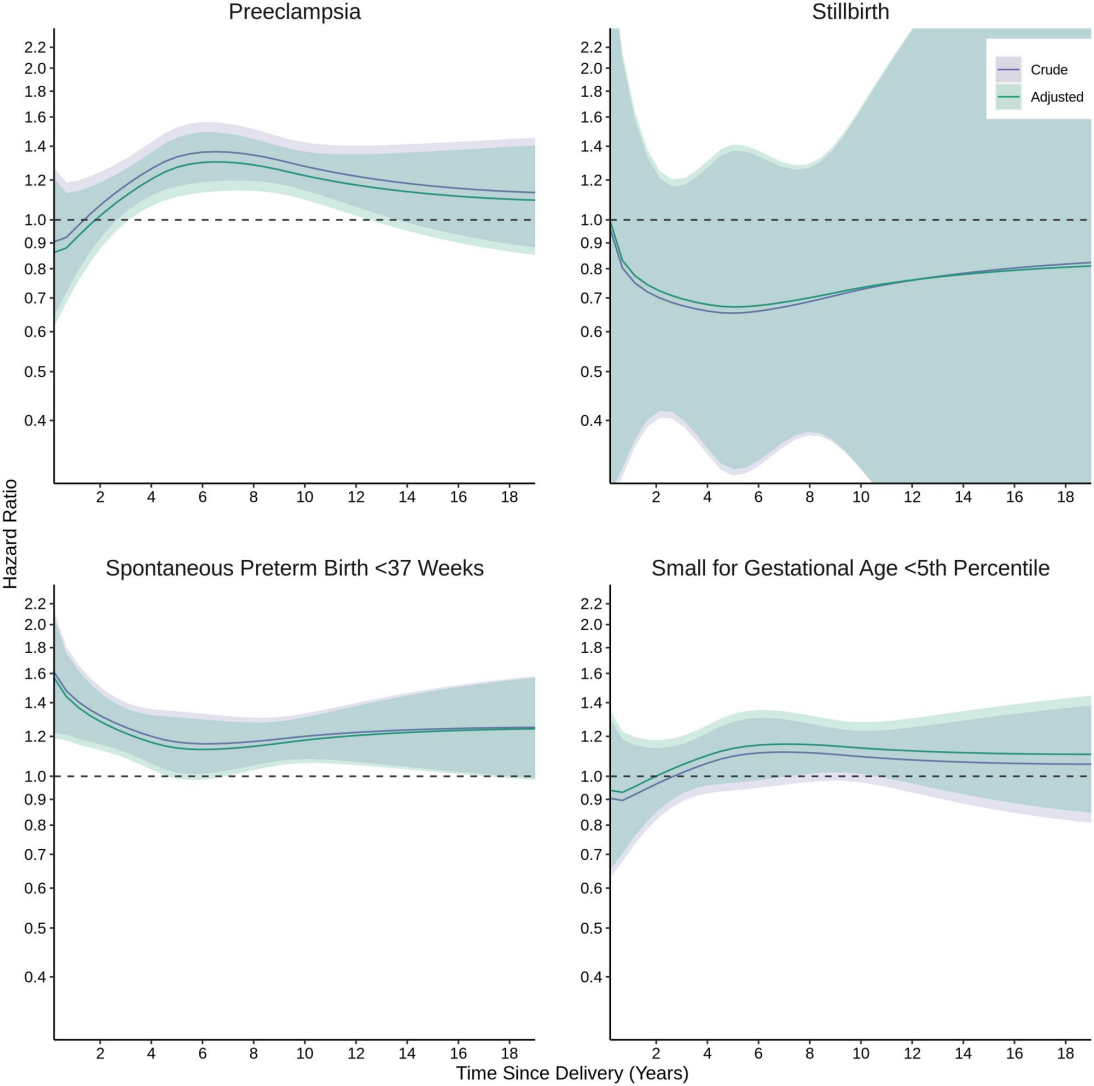
Secondary outcome: Time-dependent association of pregnancy complications and incident rheumatoid arthritis. Adjusted models controlled for calendar year, maternal age at delivery, parity, neighbourhood income quintile, rural residence and medical and psychiatric comorbidities

**Figure 5.**
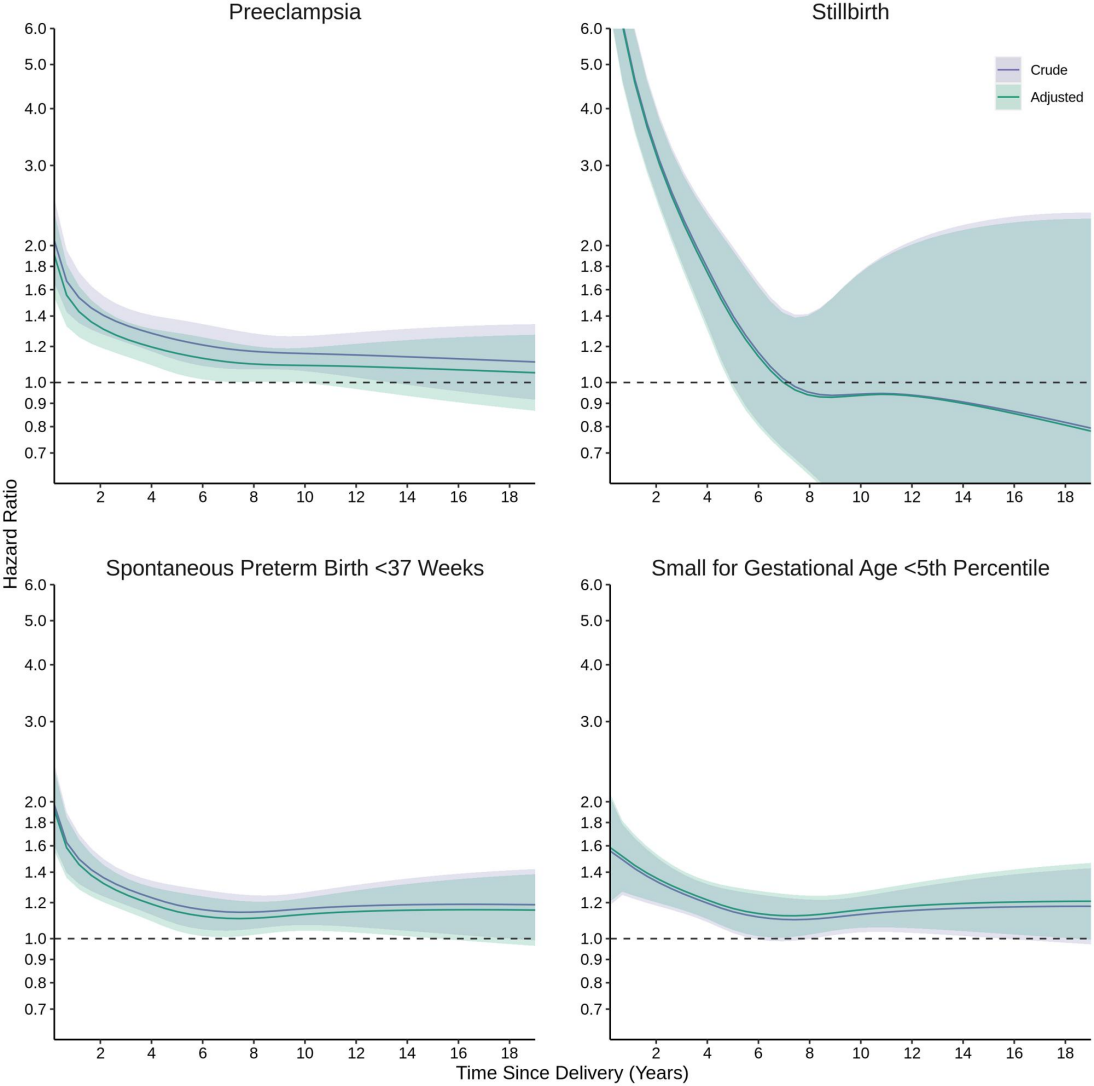
Secondary outcome: Time-dependent association of pregnancy complications and incident systemic autoimmune rheumatic disease. Adjusted models controlled for calendar year, maternal age at delivery, parity, neighbourhood income quintile, rural residence and medical and psychiatric comorbidities

The cumulative incidence of coeliac disease, rheumatoid arthritis, multiple sclerosis and SARD was generally higher after births affected by pregnancy complications than unaffected births, with pronounced differences for SARD (Figure 3). We modelled aHRs for conditions with a cumulative incidence >1% for adequate power and clinical relevance. Associations for rheumatoid arthritis differed somewhat from the main analyses; preeclampsia was associated with incident rheumatoid arthritis in a bell-shaped parabola, with the largest aHRs between ∼4 and 12 years after birth (Figure 4). Associations for incident SARD were stronger than the main analyses, with larger point estimates and 95% CI excluding the null between birth and 5 years for all complications but particularly for stillbirth where the aHR was 4.57 (95% CI 3.54–5.89) at 1-year postpartum (Figure 5).

### Additional analyses

Results from additional analyses by pregnancy complication sub-types (Supplementary Figures S2–S5 and Table S4, available as Supplementary data at *IJE* online) and limited to female-predominant autoimmune diseases (Supplementary Figure S6, available as Supplementary data at *IJE* online) generally aligned with the main analyses, with associations stronger for early- vs late-onset preeclampsia; spontaneous preterm birth with vs without PPROM, and severe SGA <3rd vs 3rd to <5th percentile. Results were robust to sensitivity analyses excluding autoimmune thyroid diseases (Supplementary Figure S7, available as Supplementary data at *IJE* online) and implementing a 365-day washout period (Supplementary Figure S8, available as Supplementary data at *IJE* online). Effect modification by antepartum haemorrhage was evident for preeclampsia (Supplementary Figure S9, available as Supplementary data at *IJE* online) and spontaneous preterm birth (Supplementary Figure S10, available as Supplementary data at *IJE* online), but not severe SGA (Supplementary Figure S11, available as Supplementary data at *IJE* online). Effect modification by parity was evident for preeclampsia with pronounced HR curves for multiparous women (Supplementary Figure S12, available as Supplementary data at *IJE* online). In the BORN sub-cohort, estimates did not change with additional adjustments for prenatal health factors (Supplementary Figure S13, available as Supplementary data at *IJE* online).

## Discussion

In this population-based study with nearly two decades of follow-up, preeclampsia, stillbirth and spontaneous preterm birth but not severe SGA were associated with modestly higher incidence of new-onset autoimmune disease in females. Risks were most elevated within the initial 3 years after birth and plateaued but generally remained elevated as individuals aged. These data suggest pregnancy complications could play a female-specific aetiologic role in the short-term, and possibly long-term, onset of autoimmune disease after childbirth. Findings indicate that females who experience pregnancy complications may benefit from greater healthcare provider awareness of autoimmune symptoms in the initial years following birth and that the periconceptional period may be an under-recognized opportunity for disease prevention.

This study’s findings converge with those of two systematic reviews on pregnancy complications and subsequent autoimmune disease.^10^^,^^11^ The first review examined the risk of adverse outcomes in pregnancies occurring before vs after autoimmune rheumatic disease diagnosis or vs the general population in 27 studies.^10^ The authors reported an increased risk of adverse pregnancy outcomes in pre-disease pregnancies that was thought to be attributable to subclinical disease activity impacting prenatal health. The second review examined the risk of incident autoimmune diseases following pregnancies with vs without complications in 25 studies.^11^ The authors reported adjusted risk ratios of 1.61 (95% CI 0.98–2.65) for preeclampsia, 2.18 (95% CI 0.65–7.34) for stillbirth and 2.02 (95% CI 1.16–3.52) for small fetal size and any autoimmune diseases. Our study extends the literature with a novel application of flexible parametric survival analysis to population-based data to capture variations in risk over time. This nuance has been largely overlooked due to methods that assume a uniform association over time (i.e. proportional hazards) or arbitrarily partition follow-up time.

The short-term associations we observed suggest pregnancy complications may be a trigger for autoimmune disease and particularly for SARD. For example, preeclampsia is characterized by immunologic abnormalities such as autoantibodies that activate cardiovascular signalling pathways.^9^^,^^45^ Similarly, despite heterogeneity in spontaneous preterm birth phenotypes, inflammation and infection are well-established risk factors with evidence of localized (e.g. placental tissue, amniotic fluid) and systemic (e.g. maternal blood) immune activation.^8^ These events are occurring when the body is undergoing the ‘stress test’ of pregnancy, maintaining a finely tuned inflammatory profile to protect from pathogens while tolerating the genetically dissimilar fetus,^46^ and may be especially vulnerable to underlying susceptibility towards immune dysfunction. Reverse causation is also possible given the multi-year latent period for autoimmune diseases.^47^ Specifically, subclinical autoimmune features predating conception may increase the likelihood of pregnancy complications,^10^^,^^48^^,^^49^ yet only become clinically apparent after birth. The long-term associations we observed additionally imply that pregnancy complications could increase women’s susceptibility to autoimmune diseases later in life. For example, preeclampsia has been linked with fetal microchimerism,^50^ the persistence of fetal cells in the maternal circulation decades after delivery,^44^ which could increase the propensity for autoimmune activity. Although the exact role of fetal microchimerism in disease progression is uncertain, our finding of an exacerbated long-term risk for autoimmune disease in females with the combined presence of preeclampsia and antepartum haemorrhage aligns with a fetal microchimerism hypothesis. Alternatively, our findings may reflect shared risk factors, such as psychosocial stress or genetic predisposition,^51–54^ or overlapping immune-mediated etiopathologies between pregnancy complications and autoimmune disease that result in their co-occurrence.

Several limitations should be considered. Principally, our study is subject to non-differential misclassification bias due to reliance on diagnostic and procedure coding in health administrative data. We were unable to conduct quantitative bias analysis due to limitations of existing methods wherein negative (impossible) cell counts frequently arise with rare exposure prevalence and even small reductions in specificity,^55^ and lack of accessible methods that extend to a time-to-event framework for correcting both binary outcomes and continuous person-time. The primary outcome of composite autoimmune disease relied on acute care encounters, which is not uncommon in epidemiologic studies on this topic,^56–59^ but would have missed milder disease presentations or cases diagnosed exclusively or earlier in outpatient settings. For example, among autoimmune disease cases identified with the secondary outcome validated algorithm approach, ∼60–95% were first diagnosed through outpatient care and 10–50% were also captured using the primary outcome acute care approach with this degree of overlap varying by disease. Accordingly, the incidence rates and timing of diagnoses we reported are conservative and associations may not generalize to less severe autoimmune disease. Residual confounding from variables that we lacked such as individual-level income and health behaviours is also possible.

Our findings signal the need for greater healthcare provider awareness of pregnancy complications as a risk factor for maternal autoimmune disease. Although autoimmune diseases are rare with a peak annual incidence in our study of ∼19 per 10 000 person-years, they are difficult to diagnose, costly, and debilitating.^60^ Screening and early detection that incorporates known risk factors is a key approach to mitigating disease progression and associated disability.^47^ Importantly, our study’s effect sizes of 20–30% increased risk were only slightly lower in magnitude than the 20–60% increased risk reported for cigarette smoking, a widely recognized risk factor for autoimmune disease.^61–63^ History and recency of pregnancy complications should therefore be considered with other risk factors in females presenting with rheumatic symptoms to facilitate timely investigation of possible autoimmune disease. Additionally, the periconceptional period when women are highly engaged with healthcare may be an untapped opportunity for autoimmune disease prevention. Future research incorporating preconception data on autoimmune biomarkers or symptoms in population-based samples would be valuable for clarifying the causal and public health significance of pregnancy complications in clinical autoimmune disease.

## Conclusion

This population-based study of 1.7 million births found pregnancy complications were associated with elevated incidence of maternal autoimmune disease in the 19 years after delivery. Risks were greatest for preeclampsia, stillbirth and spontaneous preterm birth within 3 years following delivery, and often plateaued but persisted thereafter. History of pregnancy complications may be an important female-specific risk factor to consider in clinical assessments of individuals with possible autoimmune disease to facilitate timely detection and treatment.

## Ethics approval

The study was approved by the University of Toronto Research Ethics Board (Protocol No. 43489).

## Supplementary Material

dyae115_Supplementary_Data

## Data Availability

The dataset from this study is held securely in coded form at ICES. Although data sharing agreements prohibit ICES from making the dataset publicly available, access might be granted to those who meet prespecified criteria for confidential access, available at www.ices.on.ca/DAS.
