# Supplementary material for: Pregnancy complications and new-onset maternal autoimmune disease
Source: Int J Epidemiol. 2024 Aug 27;53(5):dyae115. doi: 10.1093/ije/dyae115 (PMC11349189; doi:10.1093/ije/dyae115)
Supplement: dyae115_Supplementary_Data [file dyae115_supplementary_data.docx]

**Supplementary Material:** Scime et al. Pregnancy complications and new-onset maternal autoimmune disease: A population-based longitudinal study

Table S1. ICES databases used in the current study

Table S2. Variables, diagnostic and procedure codes, and data sources used to measure pregnancy complications

Table S2. Diagnostic codes used to measure autoimmune diseases in acute care encounters

Table S4. Time-dependent adjusted association of pregnancy complications and incident autoimmune disease, including subanalyses by complication timing, type, and/or severity: Summary of hazard ratios

Table S5. Time-dependent adjusted association of pregnancy complications and incident autoimmune disease on the additive scale: Summary of incidence rate differences per 10,000 person-years and 95% confidence intervals

Figure S1. Flowchart of study cohort

Figure S2. Subgroup analysis: Time-dependent association of preeclampsia timing and incident autoimmune disease

Figure S3. Subgroup analysis: Time-dependent association of spontaneous preterm birth timing and incident autoimmune disease

Figure S4. Subgroup analysis: Time-dependent association of spontaneous preterm birth type and incident autoimmune disease

Figure S5. Subgroup analysis: Time-dependent association of small for gestational age severity and incident autoimmune disease

Figure S6. Subgroup analysis: Time-dependent association of pregnancy complications and incident female-predominant autoimmune disease

Figure S7. Sensitivity analysis: Time-dependent association of pregnancy complications and incident autoimmune disease excluding thyroid diseases

Figure S8. Sensitivity analysis: Time-dependent association of pregnancy complications and incident autoimmune disease with a 365-day washout period since delivery

Figure S9. Sensitivity analysis: Time-dependent association of preeclampsia, antepartum hemorrhage, and incident autoimmune disease

Figure S10. Sensitivity analysis: Time-dependent association of spontaneous preterm birth, antepartum hemorrhage, and incident autoimmune disease

Figure S11. Sensitivity analysis: Time-dependent association of severe small for gestational age, antepartum hemorrhage, and incident autoimmune disease

Figure S12. Sensitivity analysis: Time-dependent association of pregnancy complications, parity, and incident autoimmune disease

Figure S13. Sensitivity analysis: Time-dependent association of pregnancy complications and incident autoimmune disease in the BORN subcohort

Table S1. ICES databases used in the current study

| Data source | Abbreviation | Data held | Inception |
| --- | --- | --- | --- |
| Health Services |  |  |  |
| Canadian Institute for Health Information Discharge Abstract Database | CIHI DAD | Hospital admissions and discharges, including obstetric deliveries in the MOMBABY dataset  Diagnostic and procedure data were coded from 1988–2001 using the ICD 9^th^ Revision & CCP and from 2002–Present using the ICD 10^th^ Revision, Canada & CCI | 1988 |
| National Ambulatory Care Reporting System & Same Day Surgery Database | NACRS & SDS | Emergency department visits and day surgery  Diagnostic and procedure data were coded from 1988–2001 using the ICD 9^th^ Revision & CCP and from 2002–Present using the ICD 10^th^ Revision, Canada & CCI | 2002 |
| Ontario Health Insurance Database | OHIP | Outpatient physician visits  Diagnostic data were coded using 3-digit physician billing codes, based on ICD 9^th^ Revision | 1991 |
| Ontario Mental Health Reporting System | OMHRS | Psychiatric hospital admissions  Diagnostic data are coded using the Diagnostic and Statistical Manual of Mental Disorders | 2005 |
| Population & Demographics |  |  |  |
| Ontario Registrar General Database | ORGD | Vital statistics on deaths | 1990 |
| Registered Persons Database | RPDB | Individual health card number (unique identifier), date of birth, sex, postal code, and death date (where applicable) | 1991 |
| Statistics Canada Census data | CENSUS | Income quintile and rural residence, defined according to postal code | 1991 |
| Acquired Datasets |  |  |  |
| Better Outcomes Registry Network | BORN | Maternal, perinatal, and newborn health, including prenatal and obstetric care | 2006 |

ICD: International Classification of Diseases and Related Health Problems. CCP: Canadian Classification of Diagnostic, Therapeutic, and Surgical Procedures. CCI: Canadian Classification of Health Interventions

Table S2. Variables, diagnostic and procedure codes, and data sources used to measure pregnancy complications

| Complication | Construct | Definition | Variables or Codes | Data Sources |
| --- | --- | --- | --- | --- |
| Preeclampsia | Any | ≥ 1 hospitalization or emergency department visit or ≥ 2 physician visits for preeclampsia between 20 weeks gestation and date of delivery | Diagnostic Codes  ICD-9: 642  ICD-10: O11, O14, O15 | CIHI DAD, NACRS, OHIP |
|  | Timing | (A) Early-onset (< 34 weeks)  (B) Late-onset (≥ 34 weeks) | Variables  Date of first health care encounter minus date of conception | CIHI DAD |
| Stillbirth | Any | Recorded on delivery hospitalization | Variables  Non-spontaneous stillborn: b_nonsp_stillbirth, m_nonsp_stillbirth  Spontaneous stillborn: b_sp_stillbirth, m_sp_stillbirth  Stillbirth: b_stillbirth, m_stillbirth | CIHI DAD |
|  | Type | (A) Fetal congenital anomalies recorded  (B) No fetal congenital anomalies recorded | Diagnostic Codes  Newborn record: Q00 to Q99 (excluding Q53, Q65, Q69, Q70, Q170, Q825)  Maternal record: O35.0, O35.1 | CIHI-DAD |
| Preterm birth | Any | (A) Provider-initiated: (1) induced or had a scheduled cesarean delivery, (2) did not experience preterm spontaneous labor, and (3) delivered at < 37 weeks.  (B) Spontaneous: (1) not provider-initiated, and (2) delivered at < 37 weeks | Diagnostic Codes  Preterm spontaneous labour: O60.1, O60.2, O42, O75.6  Procedure Codes  Induction: 5.AC.30  Cesarean: 5.MD.60  Variables  Gestational age at delivery: b_gestwks_del | CIHI DAD |
|  | Timing (Spontaneous) | (A) Extremely preterm (< 28 weeks) or very preterm (28 to < 32 weeks)  (B) Moderately preterm (32 to < 34 weeks) or late (34 to < 37 weeks) preterm | Variables  Gestational age at delivery: b_gestwks_del | CIHI DAD |
|  | Type | (A) Preterm premature rupture of membranes (PPROM) recorded  (B) No PPROM recorded | Diagnostic Codes  PPROM: O42 | CIHI DAD |
| Severe small for gestational age | Any | < 5^th^ birth weight percentile for infant sex and gestational age at birth | Variables  Birth weight: weight  Infant sex: b_sex  Gestational age at delivery: b_gestwks_del | CIHI DAD |
|  | Severity | (A) < 3^rd^ birth weight percentile  (B) < 5^th^ to 3^rd^ birth weight percentile | See previous | See previous |

Table S3. Diagnostic codes used to measure autoimmune diseases in acute care encounters

| Classification | Autoimmune Disease | ICD-9 | ICD-10-CA |
| --- | --- | --- | --- |
| Female-Predominant |  |  |  |
| Connective Tissue | Mixed/undifferentiated connective tissue disease | 710.8, 710.9 | M35.1, M35.8, M35.9 |
| Connective Tissue | Polymyositis/dermatomyositis | 710.3, 710.4 | M33, M36.0 |
| Connective Tissue | Rheumatoid arthritis | 714.0-714.2, 714.9 | M05, M06 |
| Connective Tissue | Sarcoidosis | 135, 321.4 | D86, G53.2, M63.3 |
| Connective Tissue | Scleroderma | 710.1 | M34 |
| Connective Tissue | Sjogren’s syndrome | 710.2 | M35.0 |
| Connective Tissue | Systemic lupus erythematosus | 710.0 | M32 |
| Endocrine | Addison’s disease | 255.4 | E27.1 |
| Endocrine | Hashimoto’s thyroiditis | 245.2 | E06.3 |
| Endocrine | Thyrotoxicosis/Grave’s disease | 242.0 | E05.0 |
| Gastrointestinal | Celiac disease | 579.0 | K90.0 |
| Gastrointestinal | Primary biliary cirrhosis | 571.6 | K74.3 |
| Nervous System | Multiple sclerosis | 340 | G35 |
| Nervous System | Myasthenia gravis | 358.0 | G70.0 |
| Other |  |  |  |
| Blood | Autoimmune haemolytic anaemia | 283.0 | D59.1 |
| Blood | Idiopathic thrombocytopenic purpura | NA | D69.3 |
| Blood | Pernicious anemia | 281.0 | D51.0 |
| Connective Tissue | Ankylosing spondylitis | 720.0 | M45 |
| Connective Tissue | Polymyalgia rheumatica | 725 | M35.3 |
| Connective Tissue | Psoriatic arthritis | 696.0 | L40.5, M07.0, M07.1, M07.2, M07.3, M09.0 |
| Connective Tissue | Systemic vasculitis | 446 | M30, M31 |
| Gastrointestinal | Autoimmune hepatitis | NA | K75.4 |
| Gastrointestinal | Crohn’s disease | 555 | K50 |
| Gastrointestinal | Ulcerative colitis | 556 | K51 |
| Nervous System | Guillain Barre syndrome | 357.0 | G61.0 |

NA: not applicable as there is no 4-digit ICD-9 code specific enough for the condition.

Table S4. Time-dependent adjusted association of pregnancy complications and incident autoimmune disease, including subanalyses by complication timing, type, and/or severity: Summary of hazard ratios and 95% confidence intervals

| Complication | 1 Year | 3 Years | 5 Years | 10 Years | 15 Years | 19 Years |
| --- | --- | --- | --- | --- | --- | --- |
| Preeclampsia |  |  |  |  |  |  |
| No preeclampsia (ref) | 1.0 | 1.0 | 1.0 | 1.0 | 1.0 | 1.0 |
| Preeclampsia | 1.22 (1.09, 1.36) | 1.16 (1.07, 1.25) | 1.14 (1.05, 1.25) | 1.13 (1.05, 1.22) | 1.13 (0.98, 1.29) | 1.12 (0.95, 1.32) |
| Timing |  |  |  |  |  |  |
| Early-onset | 1.28 (1.06, 1.56) | 1.13 (0.98, 1.31) | 1.11 (0.93, 1.31) | 1.24 (1.08, 1.42) | 1.35 (1.06, 1.72) | 1.40 (1.04, 1.88) |
| Late-onset | 1.18 (1.03, 1.36) | 1.17 (1.06, 1.28) | 1.16 (1.05, 1.28) | 1.10 (1.00, 1.20) | 1.05 (0.89, 1.23) | 1.03 (0.84, 1.25) |
|  |  |  |  |  |  |  |
| Stillbirth |  |  |  |  |  |  |
| Live birth (ref) | 1.0 | 1.0 | 1.0 | 1.0 | 1.0 | 1.0 |
| Stillbirth | 1.36 (0.99, 1.85) | 1.15 (0.88, 1.51) | 1.10 (0.80, 1.53) | 1.11 (0.70, 1.76) | 1.11 (0.46, 2.67) | 1.11 (0.39, 3.15) |
|  |  |  |  |  |  |  |
| Spontaneous PTB |  |  |  |  |  |  |
| No spontaneous PTB (ref) | 1.0 | 1.0 | 1.0 | 1.0 | 1.0 | 1.0 |
| Spontaneous PTB | 1.30 (1.18, 1.44) | 1.24 (1.16, 1.33) | 1.24 (1.15, 1.35) | 1.20 (1.12, 1.28) | 1.15 (1.01, 1.32) | 1.14 (0.97, 1.33) |
| Timing |  |  |  |  |  |  |
| <28 to <32 weeks | 1.30 (1.03, 1.63) | 1.13 (0.95, 1.34) | 1.10 (0.89, 1.34) | 1.20 (1.00, 1.43) | 1.29 (0.93, 1.78) | 1.32 (0.89, 1.95) |
| 32 to <37 weeks | 1.29 (1.15, 1.44) | 1.25 (1.17, 1.35) | 1.27 (1.17, 1.39) | 1.20 (1.11, 1.29) | 1.12 (0.97, 1.30) | 1.10 (0.92, 1.30) |
| Type |  |  |  |  |  |  |
| No PPROM | 1.29 (1.14, 1.46) | 1.23 (1.12, 1.34) | 1.24 (1.12, 1.38) | 1.13 (1.03, 1.24) | 1.03 (0.86, 1.24) | 1.00 (0.80, 1.24) |
| PPROM | 1.34 (1.13, 1.57) | 1.27 (1.15, 1.41) | 1.24 (1.10, 1.40) | 1.27 (1.15, 1.41) | 1.31 (1.09, 1.58) | 1.33 (1.06, 1.66) |
|  |  |  |  |  |  |  |
| Severe SGA |  |  |  |  |  |  |
| No SGA (ref) | 1.0 | 1.0 | 1.0 | 1.0 | 1.0 | 1.0 |
| SGA | 1.14 (0.99, 1.31) | 1.05 (0.96, 1.15) | 1.00 (0.91, 1.11) | 1.05 (0.97, 1.14) | 1.11 (0.96, 1.28) | 1.12 (0.94, 1.34) |
| Severity |  |  |  |  |  |  |
| <3^rd^ percentile | 1.27 (1.06, 1.53) | 1.19 (1.06, 1.34) | 1.12 (0.99, 1.27) | 1.07 (0.96, 1.19) | 1.05 (0.86, 1.26) | 1.03 (0.82, 1.30) |
| <5^th^ to 3^rd^ percentile | 0.98 (0.80, 1.20) | 0.87 (0.76, 1.01) | 0.84 (0.72, 0.99) | 1.02 (0.90, 1.15) | 1.21 (0.98, 1.49) | 1.29 (0.99, 1.67) |

PTB: preterm birth. PPROM: preterm premature rupture of membranes. SGA: small for gestational age. Adjusted models controlled for calendar year, maternal age at delivery, neighbourhood income quintile, rural residence, birth history, pregnancy loss history, and medical and psychiatric comorbidities.

Table S5. Time-dependent adjusted association of pregnancy complications and incident autoimmune disease on the additive scale: Summary of crude incidence rate and adjusted incidence rate differences per 10,000 person-years and 95% confidence intervals

| Complication | 1 Year | | | 3 Years | | | | 5 Years | | 10 Years | | 15 Years | | 19 Years | | |  |
| --- | --- | --- | --- | --- | --- | --- | --- | --- | --- | --- | --- | --- | --- | --- | --- | --- | --- |
|  | IR | aIRD  (95% CI) | | IR | | aIRD  (95% CI) | | IR | aIRD  (95% CI) | IR | aIRD  (95% CI) | IR | aIRD  (95% CI) | IR | | aIRD  (95% CI) |  |
| Preeclampsia |  |  |  | |  | |  | |  |  |  |  |  |  | |  |  |
| No preeclampsia (ref) | 12.0 | 1.0 | 13.3 | | 1.0 | | 14.0 | | 1.0 | 14.7 | 1.0 | 14.5 | 1.0 | | 14.6 | 1.0 | |
| Preeclampsia | 15.8 | 2.0  (0.7, 3.3) | 16.6 | | 1.5  (0.7, 2.4) | | 17.5 | | 1.5  (0.5, 2.5) | 18.2 | 1.5  (0.5, 2.5) | 17.8 | 1.5  (-0.3, 3.2) | | 17.7 | 1.4  (-0.7, 3.6) | |
|  |  |  |  | |  | |  | |  |  |  |  |  | |  |  | |
| Stillbirth |  |  |  | |  | |  | |  |  |  |  |  | |  |  | |
| Live birth (ref) | 12.2 | 1.0 | 13.4 | | 1.0 | | 14.1 | | 1.0 | 14.8 | 1.0 | 14.7 | 1.0 | | 14.7 | 1.0 | |
| Stillbirth | 19.3 | 3.6  (-0.5, 7.8) | 17.9 | | 1.6  (-1.5, 4.6) | | 18.4 | | 1.1  (-2.7, 4.8) | 18.6 | 1.2  (-4.8, 7.2) | 17.7 | 1.3  (-9.8, 12.3) | | 17.3 | 1.2  (-12.2, 14.7) | |
|  |  |  |  | |  | |  | |  |  |  |  |  | |  |  | |
| Spontaneous PTB |  |  |  | |  | |  | |  |  |  |  |  | |  |  | |
| No spontaneous PTB (ref) | 11.9 | 1.0 | 13.1 | | 1.0 | | 13.8 | | 1.0 | 14.6 | 1.0 | 14.4 | 1.0 | | 14.5 | 1.0 | |
| Spontaneous PTB | 16.2 | 2.8  (1.6, 4.1) | 16.9 | | 2.4  (1.5, 3.2) | | 17.9 | | 2.5  (1.5, 3.5) | 18.1 | 2.2  (1.2, 3.1) | 17.1 | 1.8  (0.1, 3.5) | | 16.9 | 1.6  (-0.5, 3.7) | |
|  |  |  |  | |  | |  | |  |  |  |  |  | |  |  | |
| Severe SGA |  |  |  | |  | |  | |  |  |  |  |  | |  |  | |
| No SGA (ref) | 12.1 | 1.0 | 13.3 | | 1.0 | | 14.1 | | 1.0 | 14.8 | 1.0 | 14.6 | 1.0 | | 14.6 | 1.0 | |
| SGA | 13.8 | 1.2  (-0.2, 2.7) | 14.0 | | 0.5  (-0.4, 1.4) | | 14.1 | | 0.1  (-1.0, 1.1) | 15.6 | 0.6  (-0.4, 1.6) | 16.1 | 1.2  (-0.6, 2.9) | | 16.4 | 1.4  (-0.8, 3.7) | |

IR: incidence rate (crude). aIRD: adjusted incidence rate difference (adjusted). PTB: preterm birth. SGA: small for gestational age. Adjusted models controlled for calendar year, maternal age at delivery, neighbourhood income quintile, rural residence, birth history, pregnancy loss history, and medical and psychiatric comorbidities.

Figure S1. Flowchart of study cohort

**
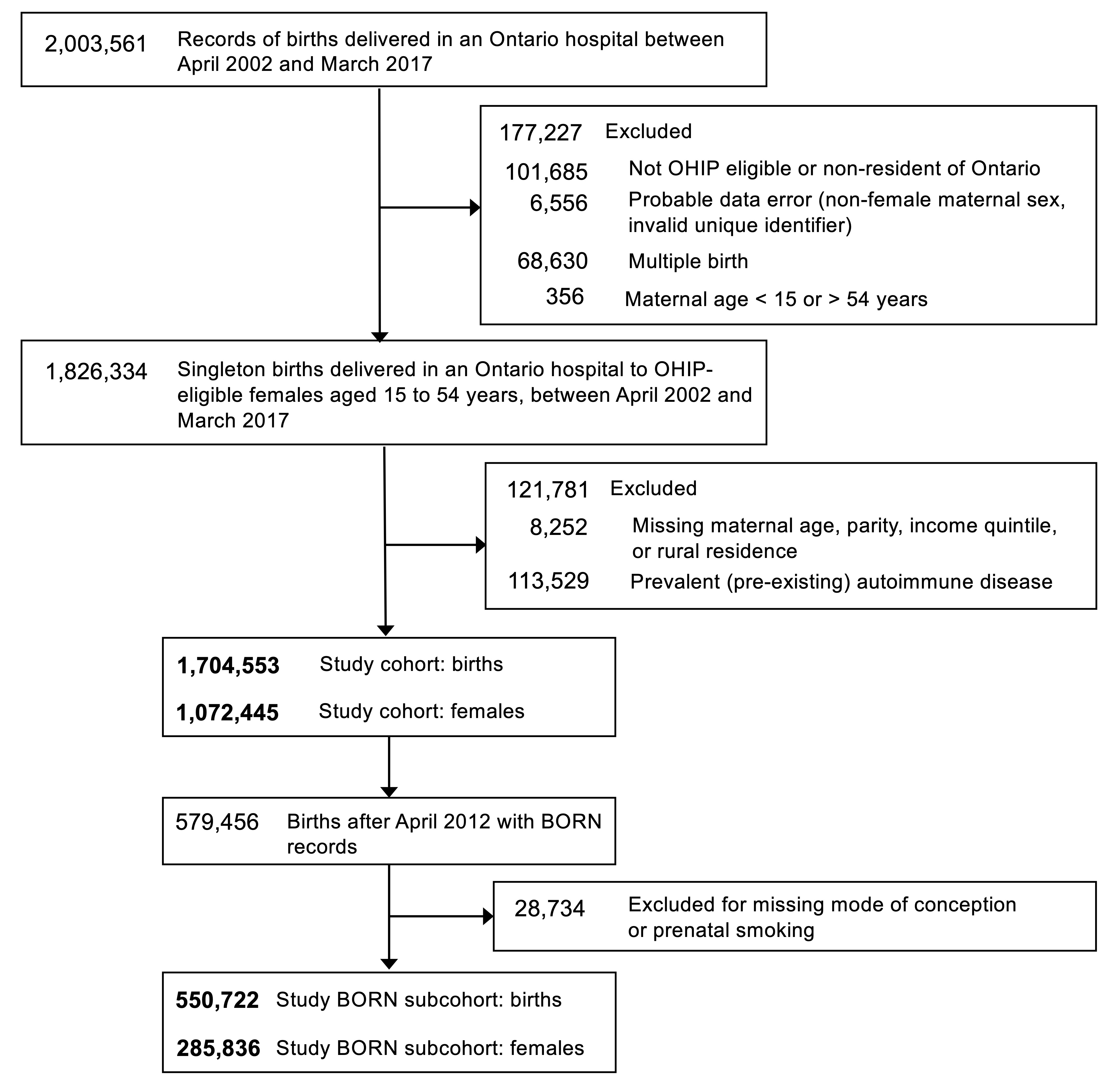
**

OHIP: Ontario Health Insurance Plan. BORN: Better Outcomes Registry & Network. Pre-existing autoimmune disease (primary outcome described in eTable 3 and validated algorithms published for celiac disease, multiple sclerosis, rheumatoid arthritis, and systemic autoimmune rheumatic disease)^31-35^ was measured from date of database inception (Table S1) to date of delivery.

Figure S2. Subgroup analysis: Time-dependent association of preeclampsia timing and incident autoimmune disease


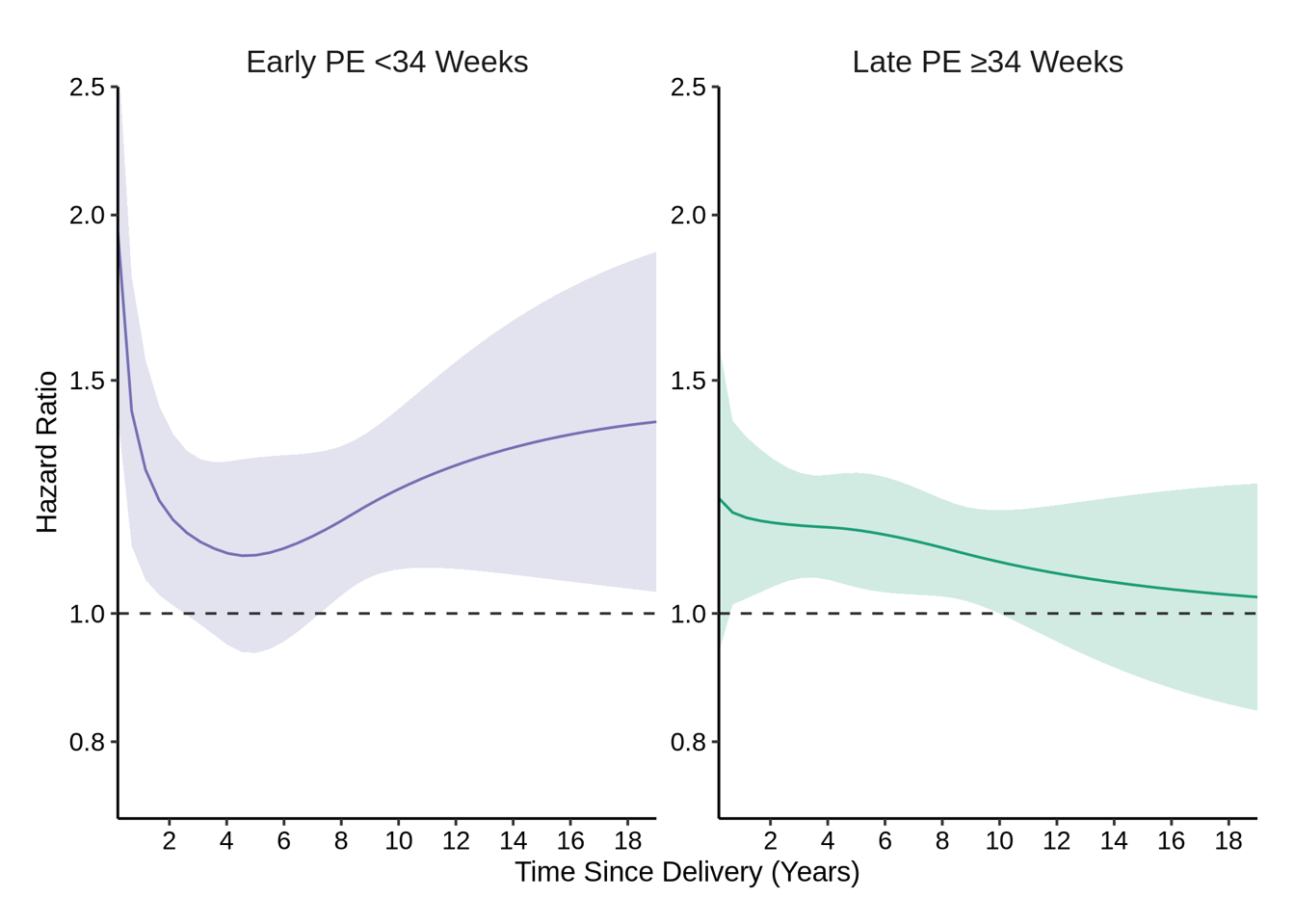


PE: preeclampsia. Models controlled for calendar year, maternal age at delivery, parity, neighbourhood income quintile, rural residence, and medical and psychiatric comorbidities.

Figure S3. Subgroup analysis: Time-dependent association of spontaneous preterm birth timing and incident autoimmune disease


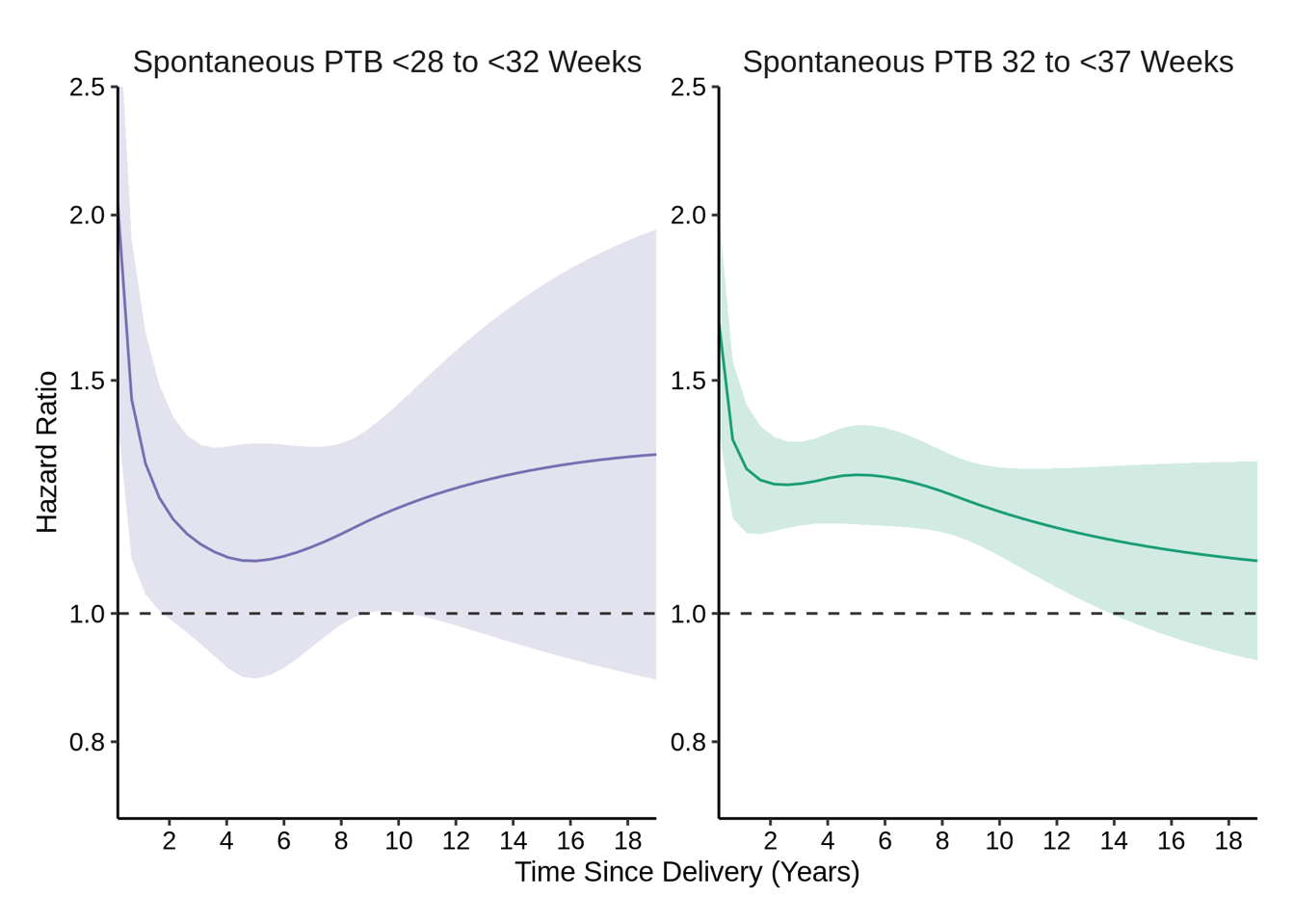


PTB: preterm birth. Models controlled for calendar year, maternal age at delivery, parity, neighbourhood income quintile, rural residence, and medical and psychiatric comorbidities.

Figure S4. Subgroup analysis: Time-dependent association of spontaneous preterm birth type and incident autoimmune disease


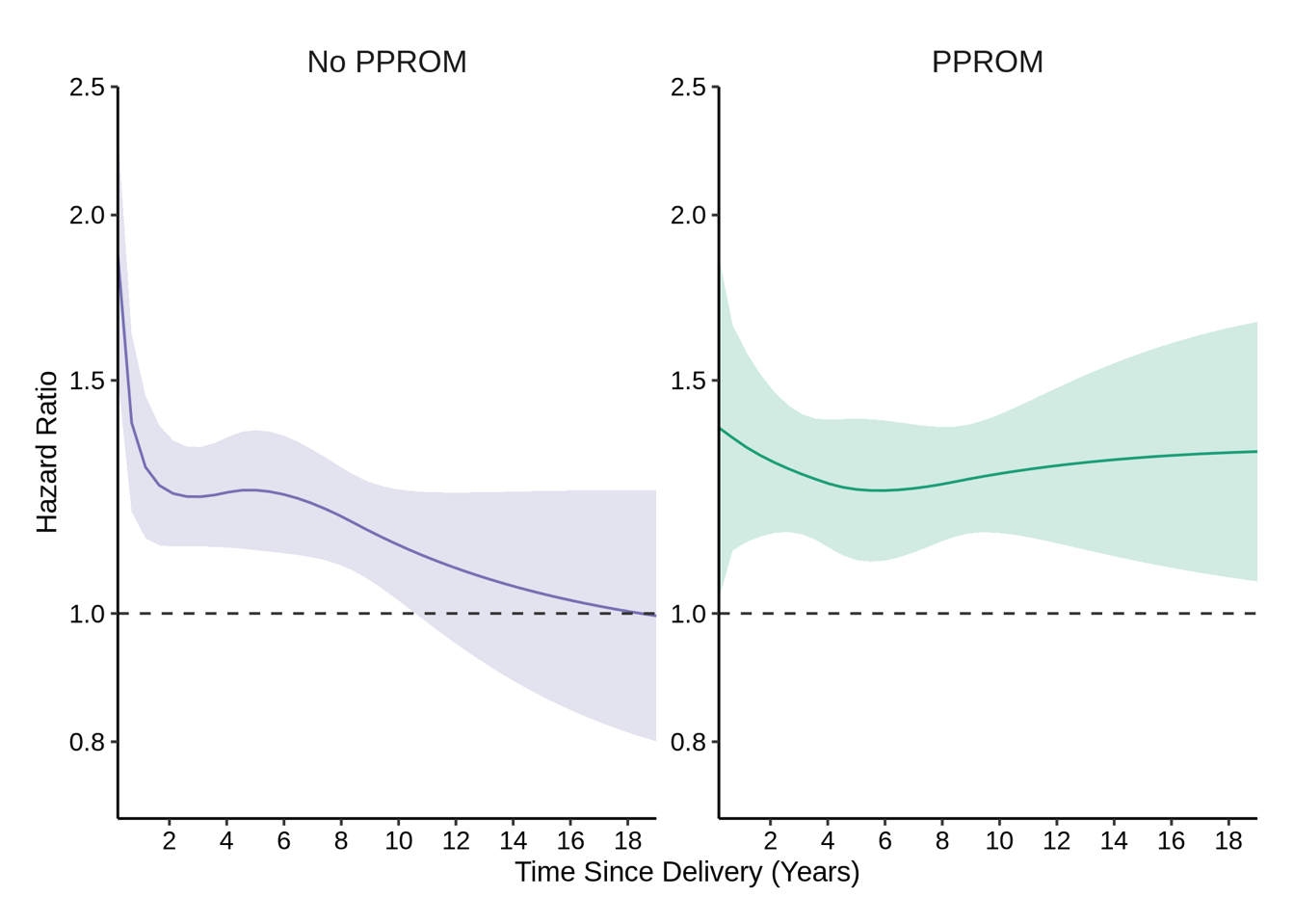


PPROM: preterm premature rupture of membranes. Models controlled for calendar year, maternal age at delivery, parity, neighbourhood income quintile, rural residence, and medical and psychiatric comorbidities.

Figure S5. Subgroup analysis: Time-dependent association of small for gestational age severity and incident autoimmune disease


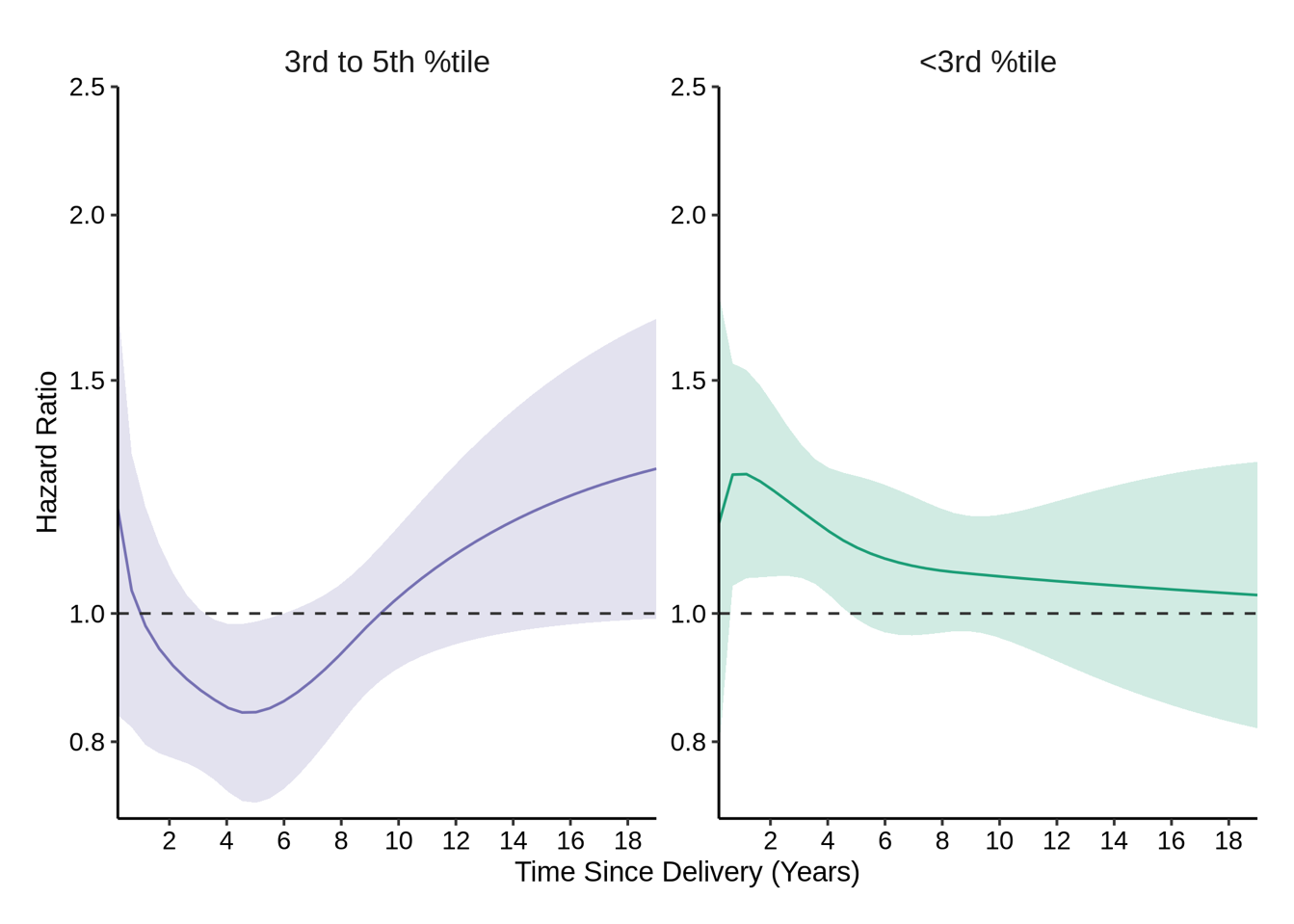


%tile: percentile. Models controlled for calendar year, maternal age at delivery, parity, neighbourhood income quintile, rural residence, and medical and psychiatric comorbidities.

Figure S6. Subgroup analysis: Time-dependent association of pregnancy complications and incident female-predominant autoimmune disease


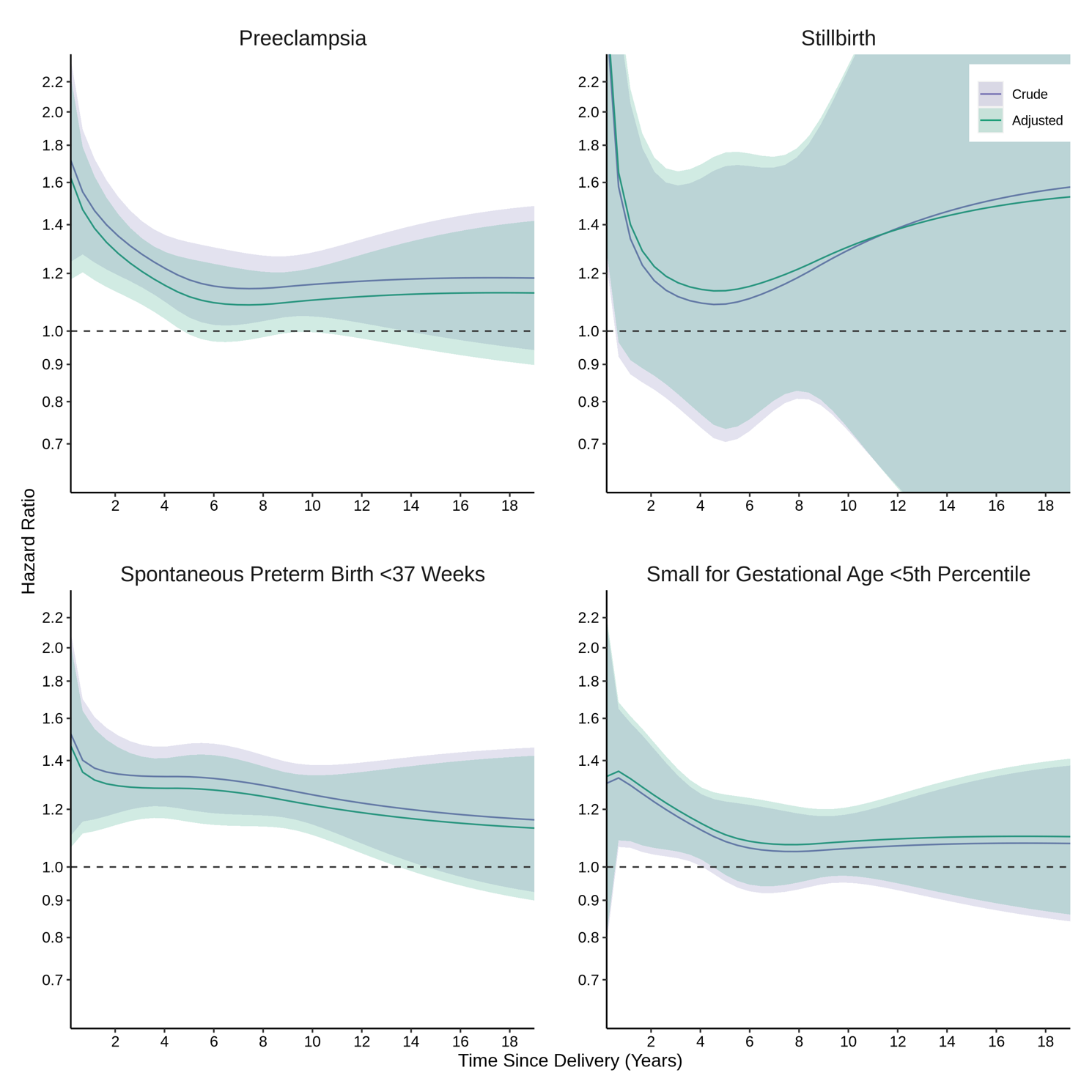


Models controlled for calendar year, maternal age at delivery, parity, neighbourhood income quintile, rural residence, and medical and psychiatric comorbidities.

Figure S7. Sensitivity analysis: Time-dependent association of pregnancy complications and incident autoimmune disease excluding thyroid diseases


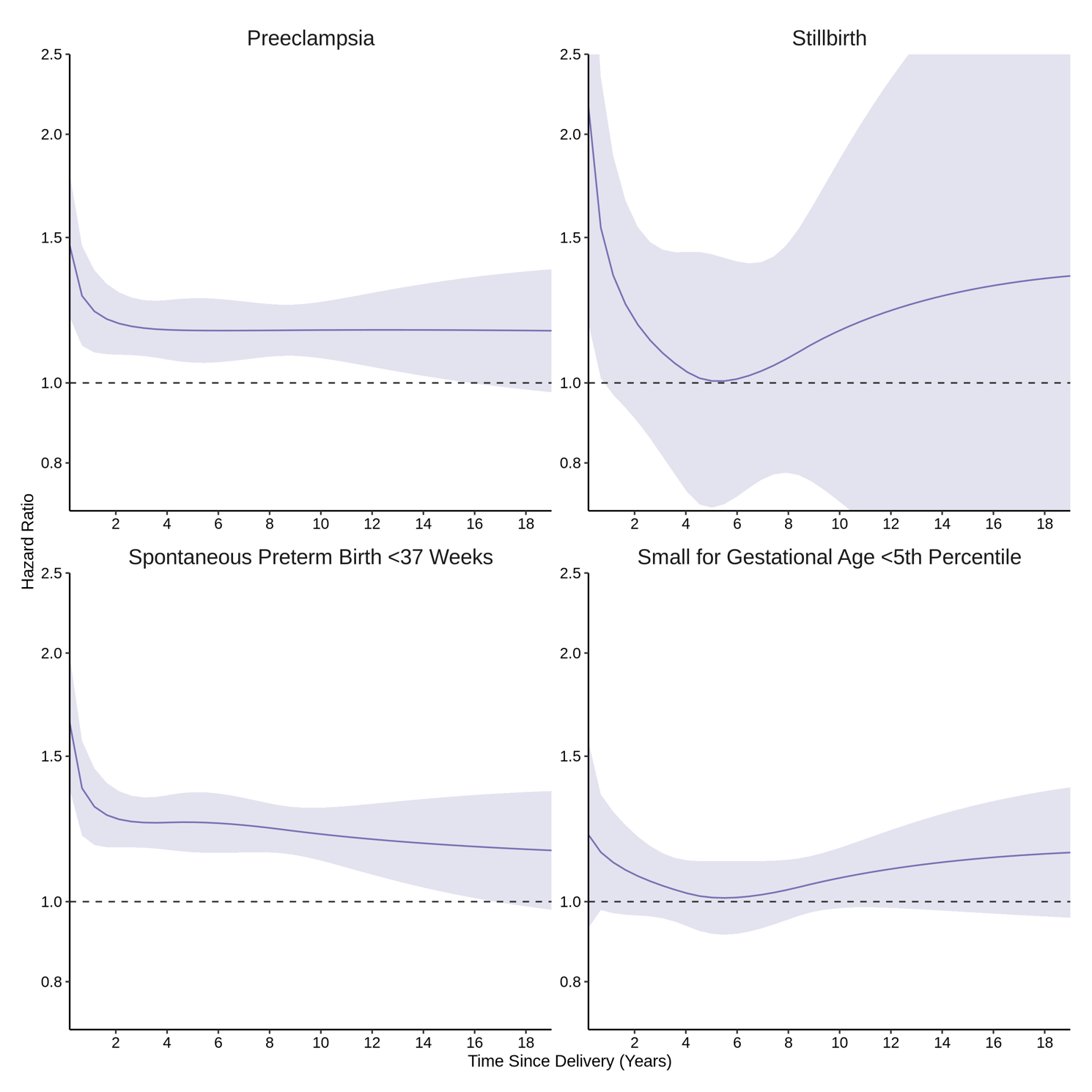


Models controlled for calendar year, maternal age at delivery, parity, neighbourhood income quintile, rural residence, and medical and psychiatric comorbidities.

Figure S8. Sensitivity analysis: Time-dependent association of pregnancy complications and incident autoimmune disease with a 365-day washout period since delivery


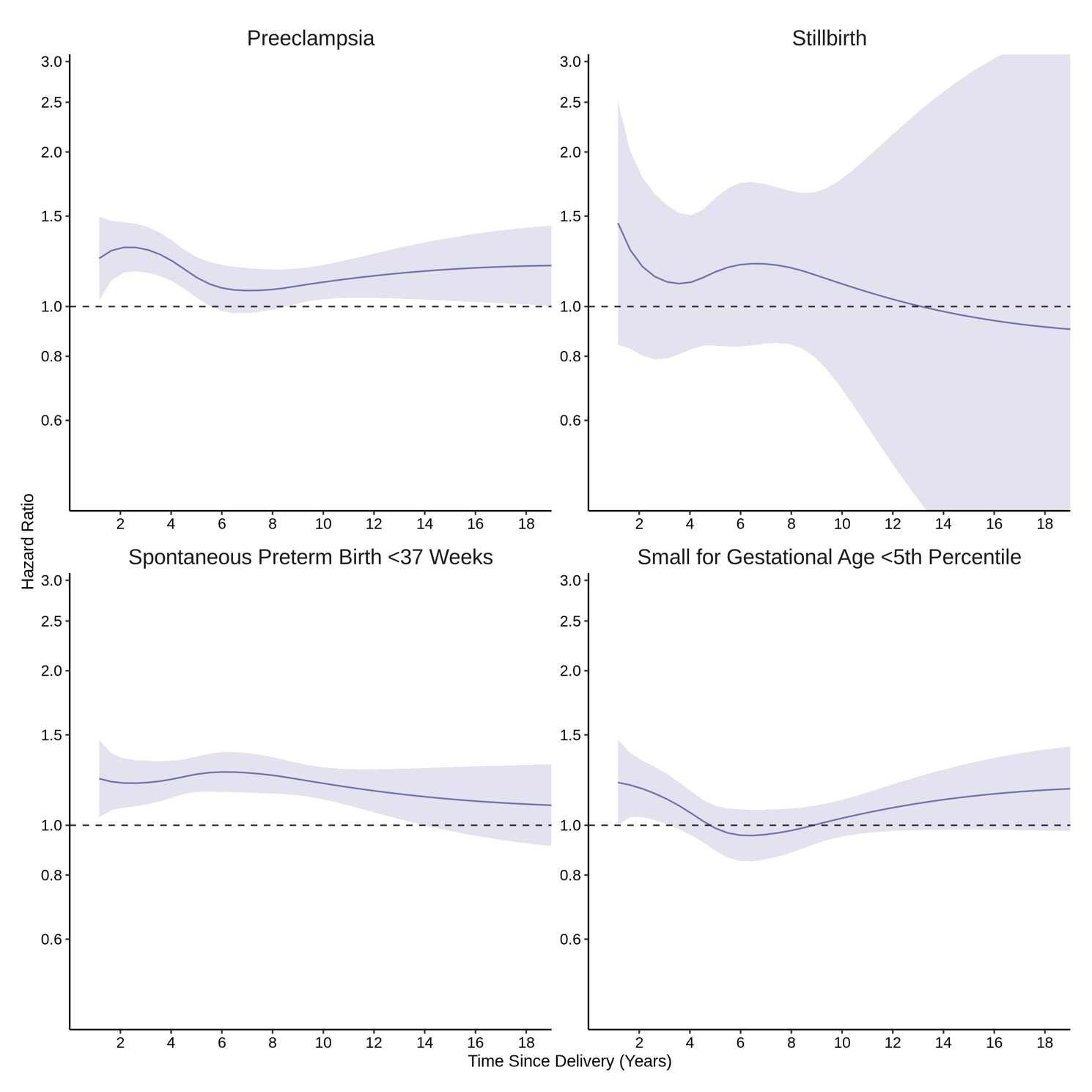


Models controlled for calendar year, maternal age at delivery, parity, neighbourhood income quintile, rural residence, and medical and psychiatric comorbidities.

Figure S9. Sensitivity analysis: Time-dependent association of preeclampsia, antepartum hemorrhage, and incident autoimmune disease


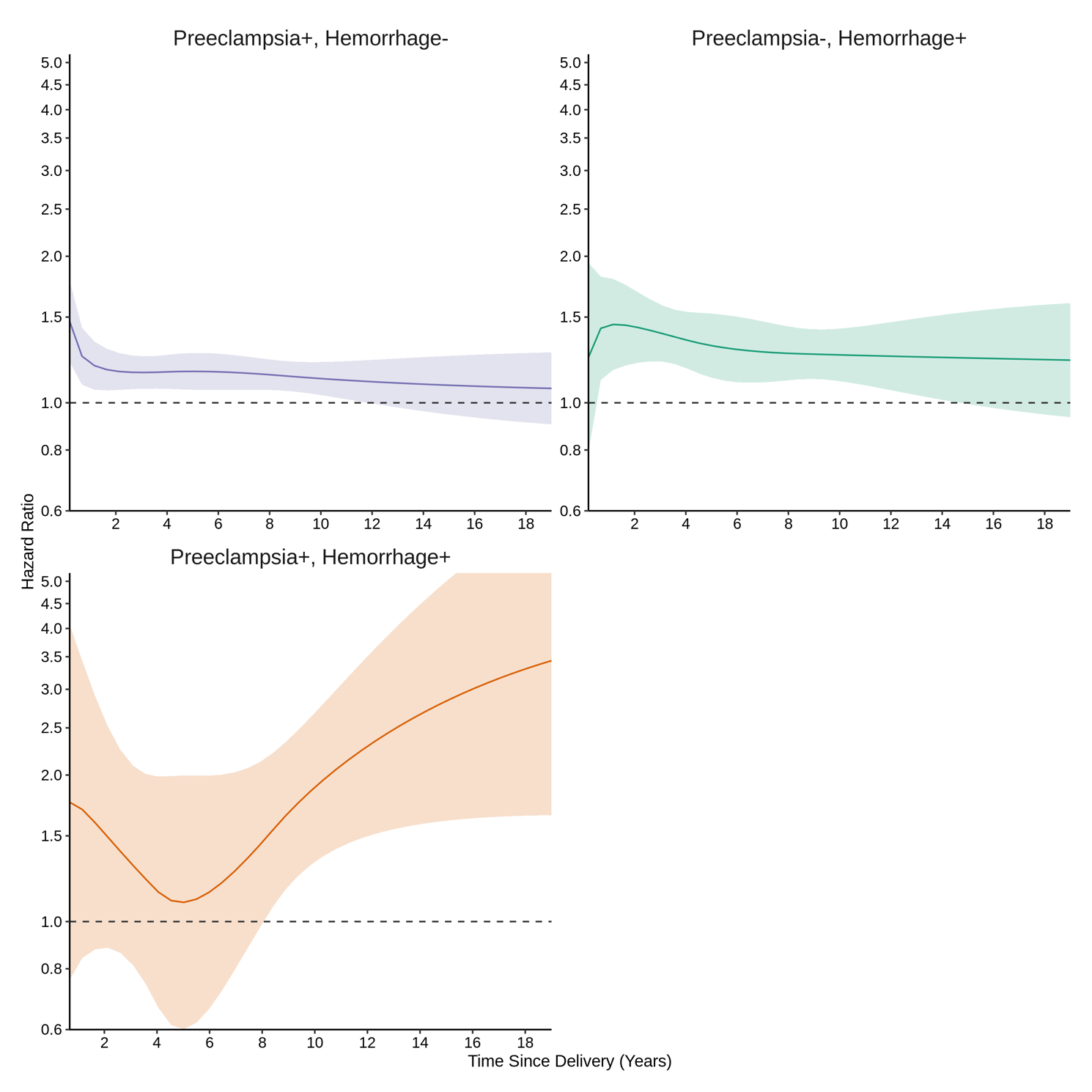


Models controlled for calendar year, maternal age at delivery, parity, neighbourhood income quintile, rural residence, and medical and psychiatric comorbidities.

Figure S10. Sensitivity analysis: Time-dependent association of spontaneous preterm birth, antepartum hemorrhage, and incident autoimmune disease


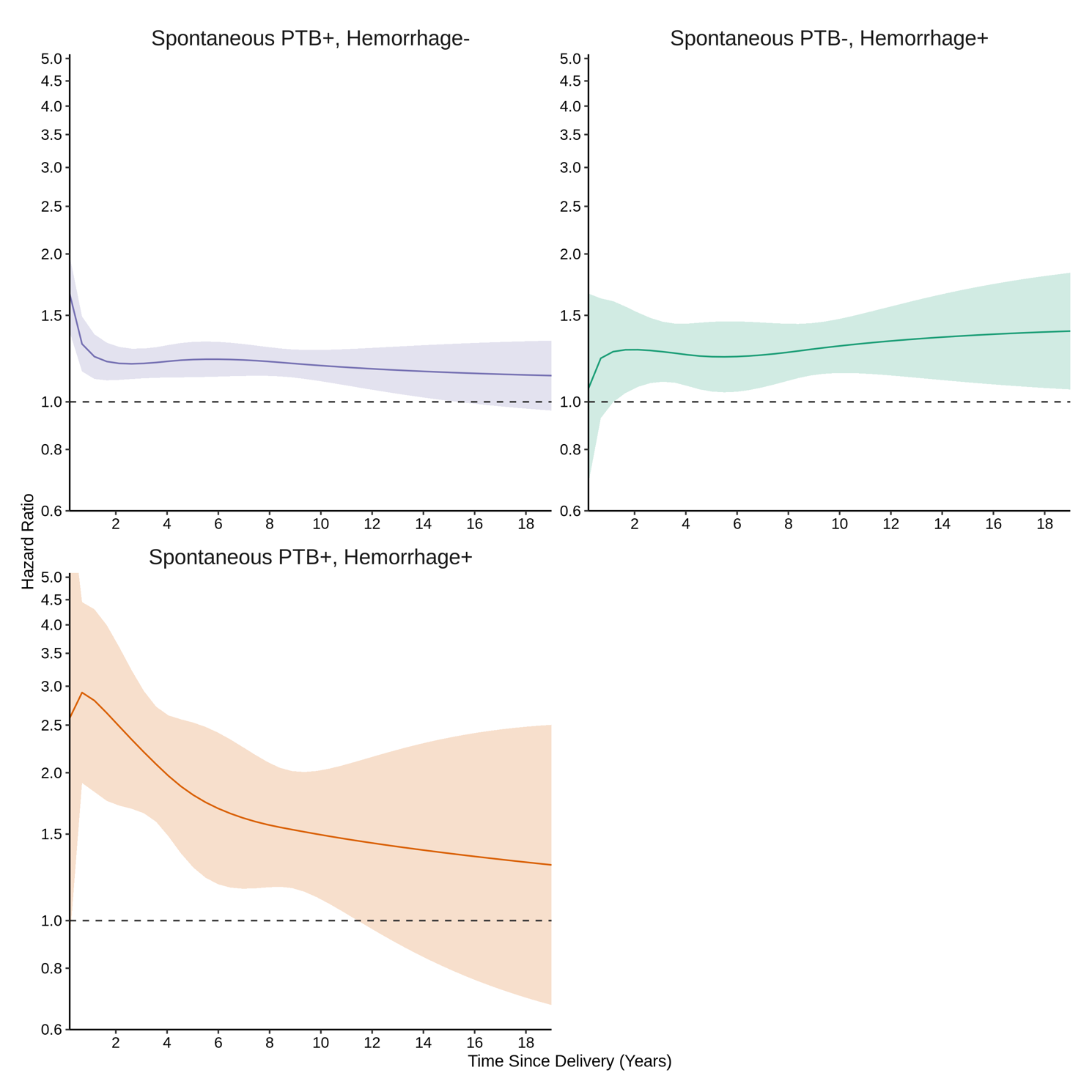


Models controlled for calendar year, maternal age at delivery, parity, neighbourhood income quintile, rural residence, and medical and psychiatric comorbidities.

Figure S11. Sensitivity analysis: Time-dependent association of severe small for gestational age, antepartum hemorrhage, and incident autoimmune disease


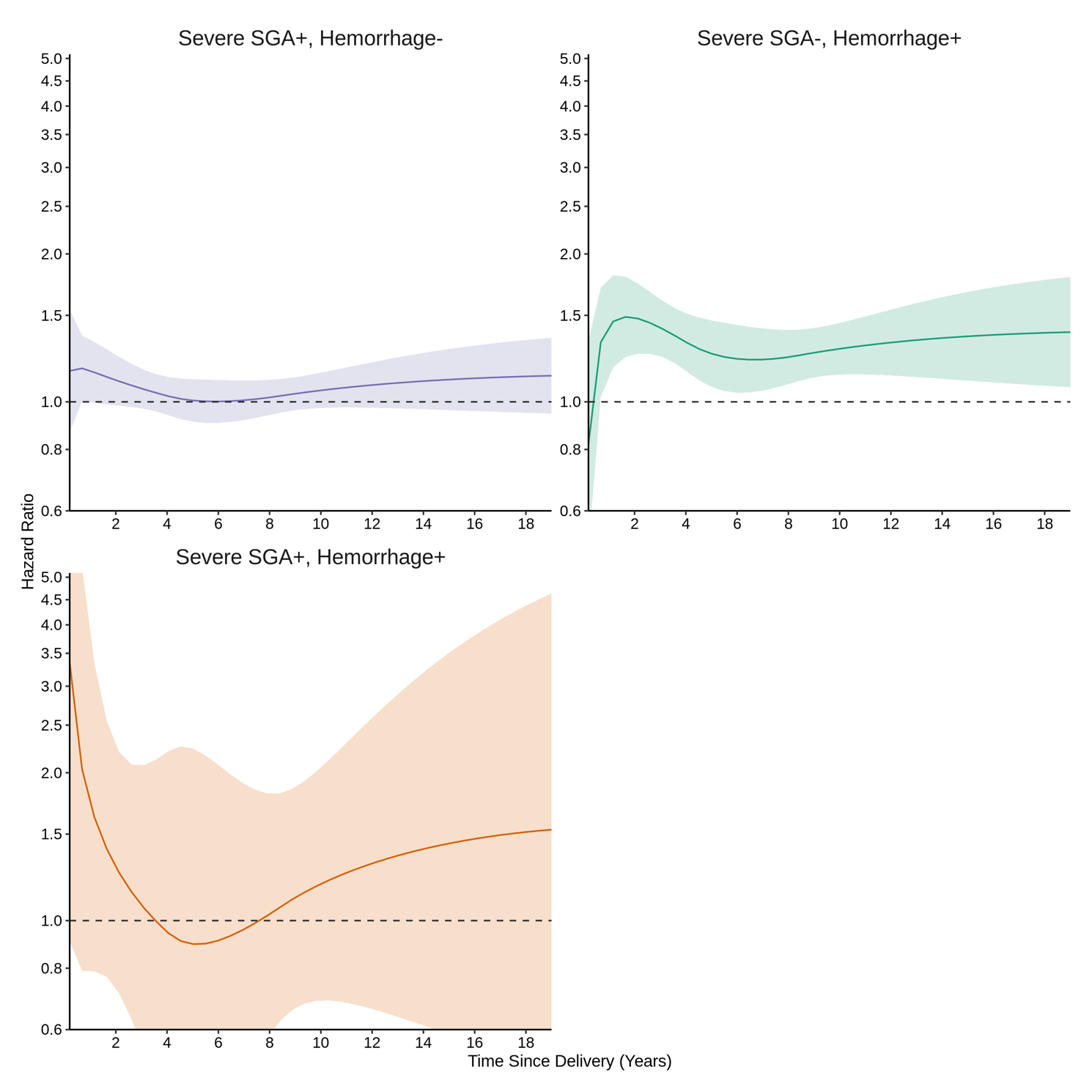


Models controlled for calendar year, maternal age at delivery, parity, neighbourhood income quintile, rural residence, and medical and psychiatric comorbidities.

Figure S12. Sensitivity analysis: Time-dependent association of pregnancy complications, parity, and incident autoimmune disease


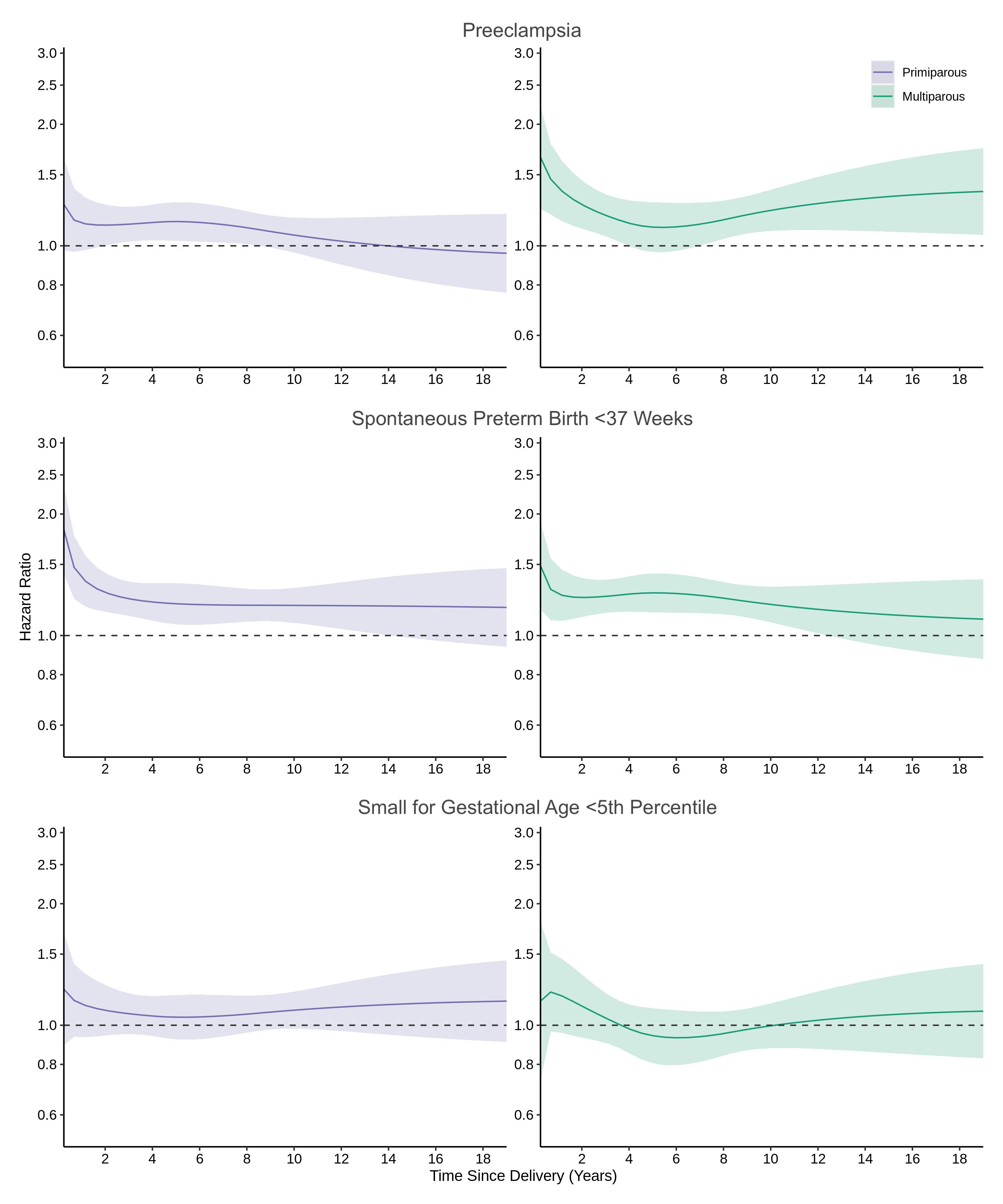


Models controlled for calendar year, maternal age at delivery, neighbourhood income quintile, rural residence, and medical and psychiatric comorbidities.

Figure S13. Sensitivity analysis: Time-dependent association of pregnancy complications and incident autoimmune disease in the BORN subcohort


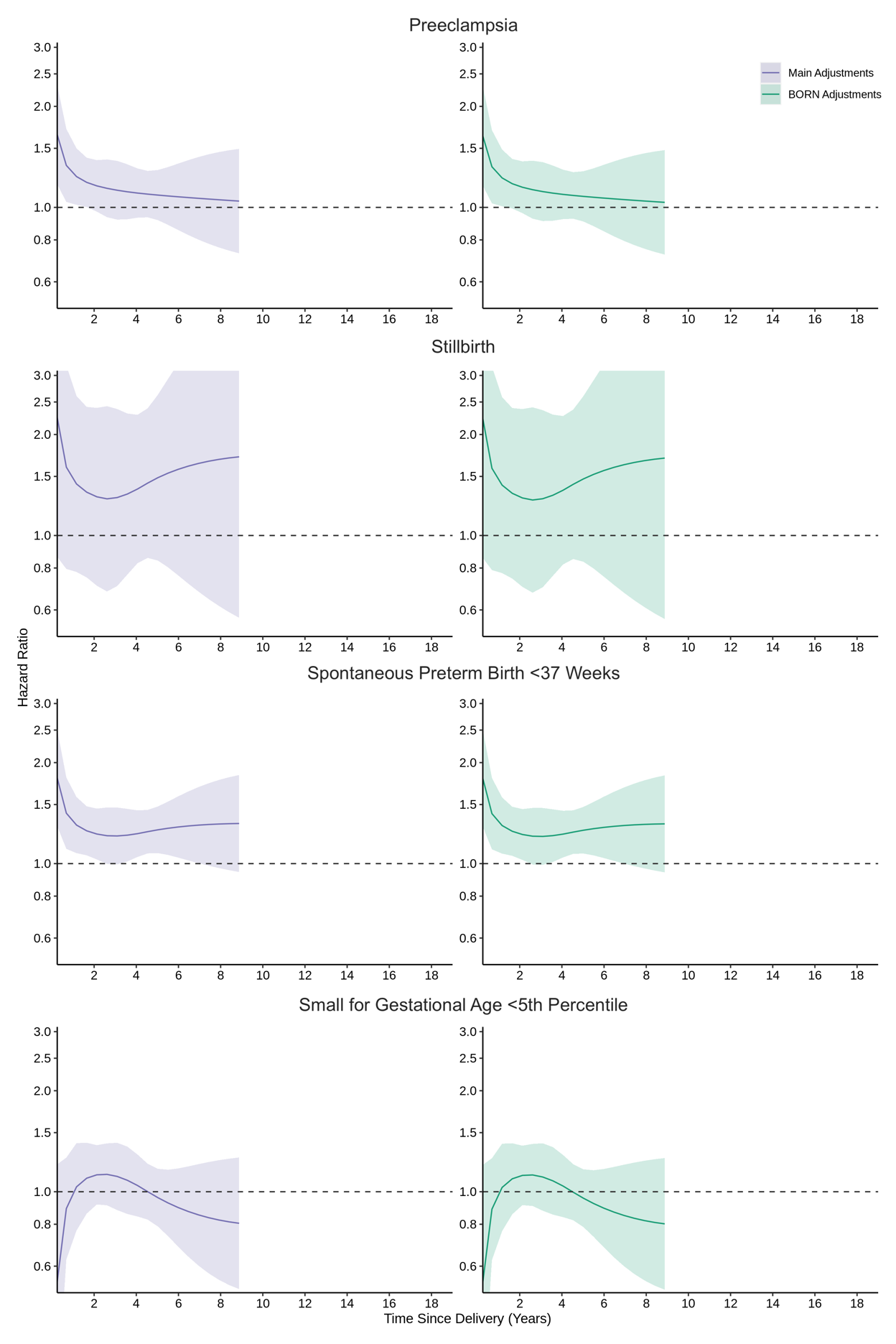


Main adjustments were calendar year, maternal age at delivery, parity, neighbourhood income quintile, rural residence, and medical and psychiatric comorbidities. BORN adjustments, on top of main adjustments, were mode of conception, maternal smoking, and pre-pregnancy obesity.
